# Balloon-occluded hepatic arterial infusion for unresectable hepatocellular carcinoma: a phase II trial interim analysis

**DOI:** 10.3389/fonc.2026.1761615

**Published:** 2026-06-01

**Authors:** Feng Li, Jian Zhang, Xiangbo An, Jianfei Liu, Benkei Li, Haiwei Li, Li Jun, Ningfang Wang, Guinan Jiang, Ye Jiang, Dehui Zhang, Fei Gao, Lei Song, Jun Zhou, Wenheng Zheng, Min Ji, Feng Wang

**Affiliations:** 1 Department of Interventional Therapy, The First Affiliated Hospital of Dalian Medical University, Dalian, Liaoning, China; 2 Department of Interventional Therapy, Dalian Public Health Clinical Center, Dalian, Liaoning, China; 3 Department of Interventional Radiology, Liaoning Cancer Hospital & Institute, Cancer Hospital of Dalian University of Technology, Cancer Hospital of China Medical University, Shenyang, Liaoning, China; 4 Department of Interventional General Surgery, Wafangdian Central Hospital, Dalian, Liaoning, China; 5 Department of Interventional Vascular Surgery, Panjin Central Hospital, Panjin, Liaoning, China; 6 Department of Interventional Therapy, The Second Affiliated Hospital of Dalian Medical University, Dalian, Liaoning, China; 7 Department of Invasive Technology, Affiliated Zhongshan Hospital of Dalian University, Dalian, Liaoning, China

**Keywords:** bHAIC, clinical study, FOLFOX, hepatocellular carcinoma, objective response rate

## Abstract

**Background:**

Hepatic arterial infusion chemotherapy (HAIC) is increasingly used to treat unresectable hepatocellular carcinoma (HCC), yet some HCC patients remain unresponsive or show disease progression.

**Purpose:**

This phase II study (ChiCTR2300077718) aimed to assess the efficacy and safety of balloon-occluded HAIC (bHAIC) based on the FOLFOX (oxaliplatin plus fluorouracil and leucovorin) regimen (bHAIC-FO) in unresectable HCC.

**Materials and methods:**

The present work is an investigator-initiated, multicenter, single-arm, prospective cohort trial. Unresectable HCC patients who had undergone a successful bHAIC procedure were included in the efficacy and safety analysis. After all patients received at least one post-bHAIC follow-up, an interim analysis was conducted in accordance with the predefined protocol. The primary endpoint of this interim analysis was the objective response rate (ORR). Secondary endpoints included changes in alpha-fetoprotein (AFP) levels and the occurrence of adverse events (AEs). Interim data were collected and analyzed using SPSS, version 27.0.

**Results:**

50 patients received at least one successful bHAIC procedure and were included in the efficacy and safety analysis. The best ORR for 45 evaluable patients was 53.3% according to Response Evaluation Criteria in Solid Tumors 1.1 (RECIST 1.1) criteria, and 82.2% according to modified RECIST (mRECIST) criteria. In the patients population, 36 treatment-naive individuals achieved an 88.9% ORR according to mRECIST criteria, with 15 cases achieving radiological complete response (CR). Overall, 77.3% of patients with elevated AFP levels experienced a reduction of over 50% in their AFP levels. All AEs were manageable by medication or dose modifications.

**Conclusion:**

bHAIC-FO demonstrates encouraging efficacy and acceptable safety profiles in unresectable HCC treatment, particularly in treatment-naive patients.

**Clinical trial registration:**

www.chictr.org.cn, identifier ChiCTR2300077718

## Introduction

Liver cancer remains a global health challenge, ranking third among the causes of cancer-related deaths with 757,948 cases in 2022 (1). Hepatocellular carcinoma (HCC) is the most common form of liver cancer, and accounts for∼90% of all cases (2). The treatment for HCC is assigned according to tumor stages. According to Barcelona Clinic Liver Cancer staging system, the main treatment methods for intermediate and advanced HCC include transarterial chemoembolization (TACE) and systemic therapy (3). Hepatic arterial infusion chemotherapy (HAIC) is widely used in Asian countries, and is recommended by multiple guidelines as a treatment option for unresectable HCC (4–7). Recently, HAIC based on the oxaliplatin plus fluorouracil and leucovorin (FOLFOX) regimen (HAIC-FO) for unresectable HCC has garnered increasing attention. The reported efficacy data indicate an objective response rate (ORR) ranging from 25.0% to 37.4%, and an overall survival (OS) ranging from 12.9 to 15.9 months (8–12). While some HCC patients treated with HAIC-FO exhibit encouraging reductions in tumor size and alpha-fetoprotein (AFP) levels, a significant number either do not respond or experience disease progression after treatment (9). Moreover, the observed OS with HAIC-FO in unresectable HCC falls below the 19.2 months reported in the Imbrave150 study (13). In light of above studies, the efficacy of HAIC-FO in treating unresectable HCC remains limited.

Despite using the same pharmacological regimen, HAIC-FO demonstrates better efficacy in HCC compared to FOLFOX chemotherapy alone (14). The enhanced tumor response observed with HAIC-FO compared to FOLFOX chemotherapy alone can be attributed to the elevated local plasma concentrations of oxaliplatin and fluorouracil. These elevated concentrations are achieved through continuous infusion into the hepatic artery, allowing for more effective targeting of tumors due to the intrahepatic first-pass effect (15, 16). In theory, the enhanced local concentration and extended exposure duration of chemotherapeutic drugs at the tumor site should amplify the anti-tumor efficacy of HAIC, especially for drugs that depend on both time and concentration for their effects (17). We originally proposed the balloon-occluded HAIC (bHAIC) by enhancing the standard HAIC protocol. This involves pre-positioning an occluding balloon in the hepatic artery prior to HAIC administration, leading to improved therapeutic outcomes in HCC through elevated local plasma drug concentrations and prolonged drug retention at the tumor site.

Here we present an interim analysis of a phase II, prospective clinical trial of bHAIC-FO in unresectable HCC. The study demonstrates that bHAIC-FO yields promising outcomes in these patients.

## Methods

### Study design and participants

We performed a phase II, multicenter, single-arm, prospective cohort trial at five hospitals in China. Efficacy was assessed by using the ORR and changes in AFP levels, while safety was assessed through adverse events (AEs) monitoring and laboratory evaluations. Anti-tumor activity was assessed according to both the Response Evaluation Criteria in Solid Tumors 1.1 (RECIST 1.1) and modified RECIST (mRECIST) criteria, with evaluation time points including baseline, every four weeks during the bHAIC-FO session, and every eight weeks thereafter. All AEs were monitored and recorded, with their relationship to bHAIC-FO treatment assessed by the study investigators, and subsequent classification by severity grade according to the common terminology criteria for adverse events 5.0 (CTCAE 5.0). Next, an interim analysis was conducted after patients had received at least one post-bHAIC follow-up to evaluate the treatment efficacy and safety. The cutoff date for this analysis was January 23, 2025.

Inclusion criteria for enrolled patients included individuals aged 18 years or older with diagnosed HCC who had a life expectancy greater than three months, had an eastern cooperative oncology group (ECOG) performance status score of 0–2, and had a Child-Pugh liver function grade A or B (C-P score ≤7). All patients met the diagnostic criteria for HCC according to the practice guidelines of the American Association for the Study of Liver Diseases.22 The inclusion criteria were as follows: unresectable hepatocellular carcinoma (locally advanced, intermediate HCC not amenable to TACE or with disease progression after two cycles of TACE; no obvious distant metastasis; adequate organ function (leukocyte count ≥3.0 × 109/L, platelet cell count ≥50 × 109/L, total bilirubin ≤1.5 times the upper limit of normal [ULN], transaminase ≤ five times the ULN, and serum creatinine ≤1.5 times the ULN)).

This study was conducted following the Declaration of Helsinki. Ethics approval was obtained from the ethical committee of all centers, and written informed consent was obtained from all participants. The study was registered at www.chictr.org.cn (Chinese Clinical Trial Registry identifier: ChiCTR2300077718). More detailed inclusion and exclusion criteria are listed in the Study Protocol (**Supplementary Material 1**).

### Interventional treatment

Patients underwent bHAIC every 4 weeks, with the regimen performed for a maximum of three cycles. Detailed bhAIC procedural protocols, pharmacological regimens, and underlying technical principles are provided in **Supplementary Material 2**.

A bHAIC procedure was successful if: (1) The balloon was positioned beyond the gastroduodenal artery; otherwise, complete embolization of the gastroduodenal artery was necessary; (2) When multiple arteries supplied the tumor, the artery occluded by the balloon should cover at least 70% of the tumor’s blood supply area.

The criteria for discontinuing bHAIC: (1) complete response (CR); (2) progressive disease (PD); (3) intolerable adverse events (AEs); (4) investigator-determined need to stop bHAIC based on patient condition. If the planned bHAIC was delayed or interrupted, the patient remained in the study and continued to receive treatment and follow-up.

### Assessments

Efficacy was assessed by using the ORR and changes in AFP levels; safety was assessed through AEs monitoring and laboratory evaluations. Anti-tumor activity was assessed according to both the Response Evaluation Criteria in Solid Tumors 1.1 (RECIST 1.1) and modified RECIST (mRECIST) criteria, with evaluation time points including baseline, every 4 weeks during the bHAIC-FO session, and every 8 weeks thereafter. Radiological evaluations were performed by local radiologists certified by the Chinese Society of Interventional Radiology. A standard operating procedure for response assessment was implemented across centers, and 10% of images were randomly cross-checked by a core lab radiologist to ensure consistency. No central review was conducted due to resource constraints, potentially introducing bias.

All AEs were meticulously monitored and recorded, with their relationship to bHAIC-FO assessed by investigators, and subsequent classification by severity grade according to the Common Terminology Criteria for Adverse Events 5.0 (CTCAE 5.0). An interim analysis was conducted after patients received ≥1 post-bHAIC follow-up.

### Outcomes and endpoints

According to the predefined study protocol (ChiCTR2300077718), the primary endpoint of this interim analysis was ORR. Secondary endpoints included changes in AFP levels, and the safety profile of bHAIC-FO. The endpoints such as duration of response, progression-free survival, and OS have been collected, but not reported here; They will be reported in the final analysis.

### Statistics

The sample size was calculated based on the following assumption: the ORR (RECIST 1.1) for conventional HAIC-FO in unresectable HCC was approximately 30% (25.0% to 37.4%) (8–12). It was anticipated that bHAIC-FO could increase the ORR from 30% to 50%. To detect this difference with 80% power and a two-sided α = 0.05, a sample size of 49 patients was required, assuming an estimated dropout rate of 10%.

As of January 23, 2025, the study was ongoing and enrolled patients had received at least one bHAIC treatment and follow-up. Interim data were collected and analyzed using SPSS, version 27.0. Variables were described using descriptive statistical terminology including counts, percentages, median with interquartile range (or mean ± standard deviation). Efficacy analyses were based on the modified intention-to-treat (mITT) patients (n = 50, completed ≥1 bHAIC) and evaluable patients (n = 45, ≥ 2 follow-ups). The interim analysis was performed according to the pre−specified criteria. Given the favorable preliminary results observed at this planned interim time point, we decided to release the data before study completion to facilitate earlier awareness and promote further investigation of this new treatment approach for a broader range of potential patients.

## Results

### Patient population

**Figure 1:**
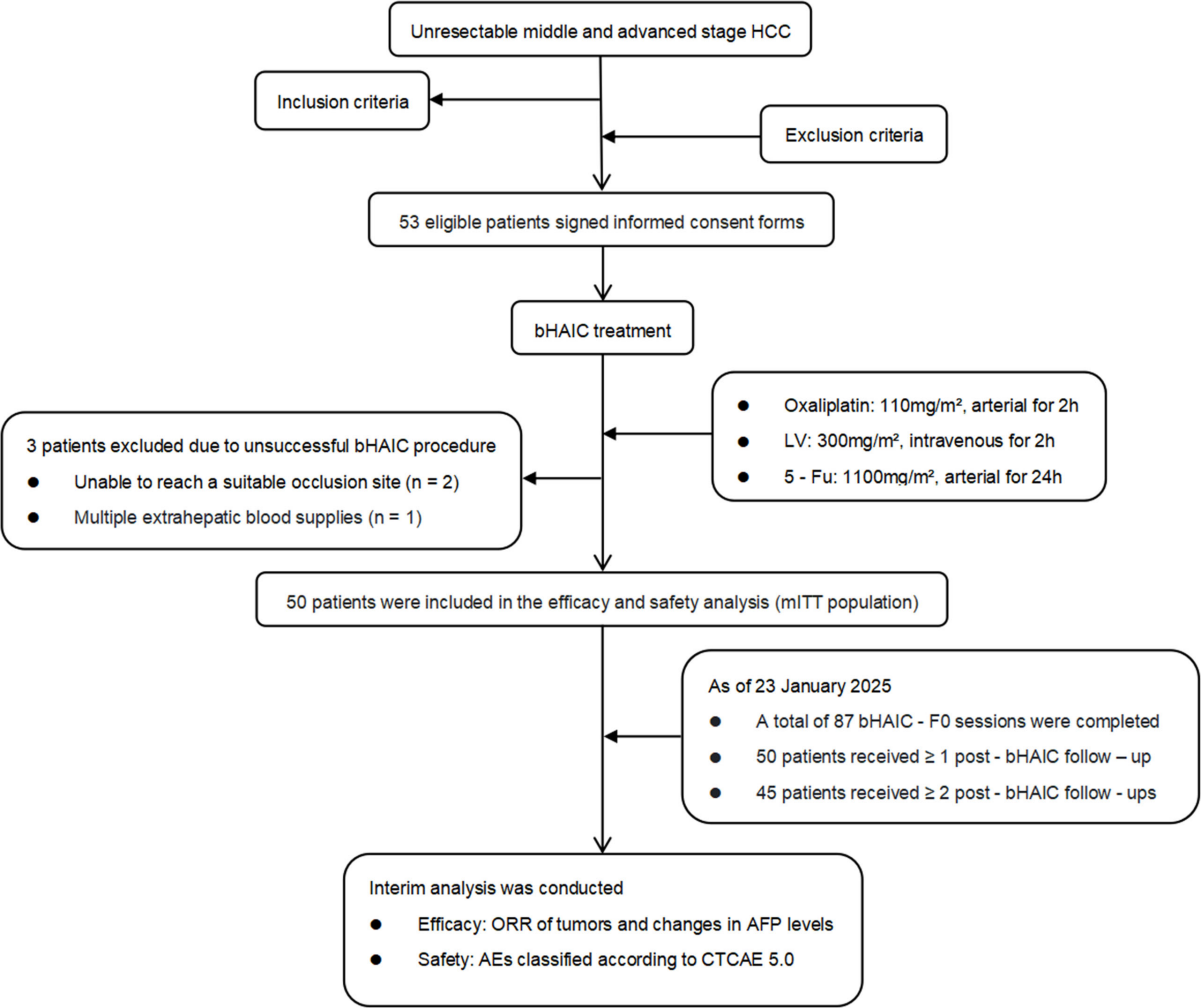
Study flowchart and interim outcomes. AEs, adverse events, bHAIC, balloon-occluded HAIC; bHAIC-FO, bHAIC based on FOLFOX regimen; CTCAE, Common Terminology Criteria for Adverse Events; mITT, modified intent-to-treatment; ORR, objective response rate.

**Figure 2:**
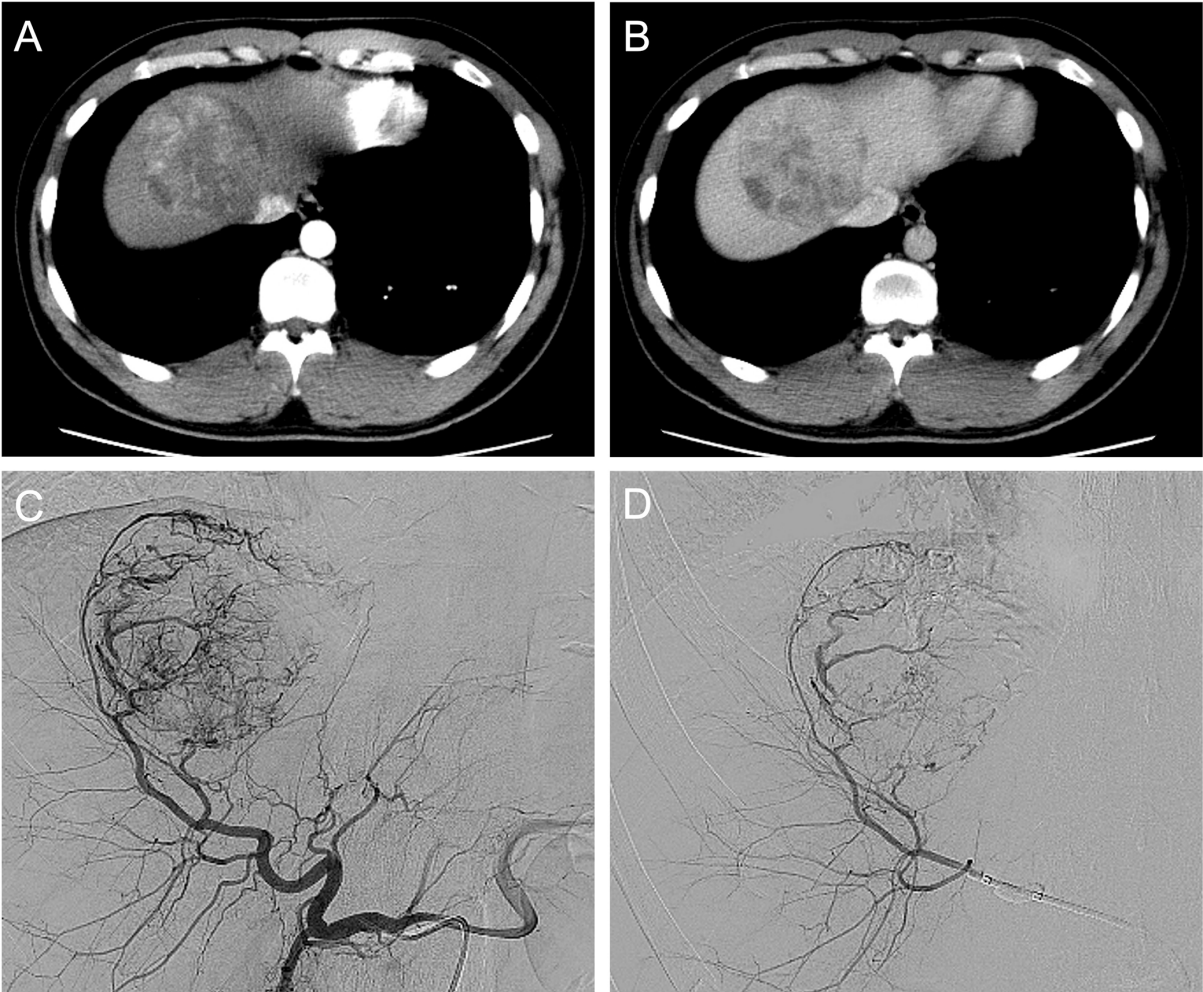
Images in a 28-year-old male with unresectable hepatocellular carcinoma (HCC) who was successfully converted to resectable status after balloon-occluded HAIC (bHAIC) and ultimately underwent radical ablation. **(A, B)** Pretreatment enhanced Magnetic Resonance (MR) shows a 77 × 74 mm lesion in segment IV with unclear boundaries from the inferior vena cava. **(C, D)** Selective arteriographic images show the large HCC **(C)** and the position of the balloon catheter occlusion **(D)**.

**Figure 3:**
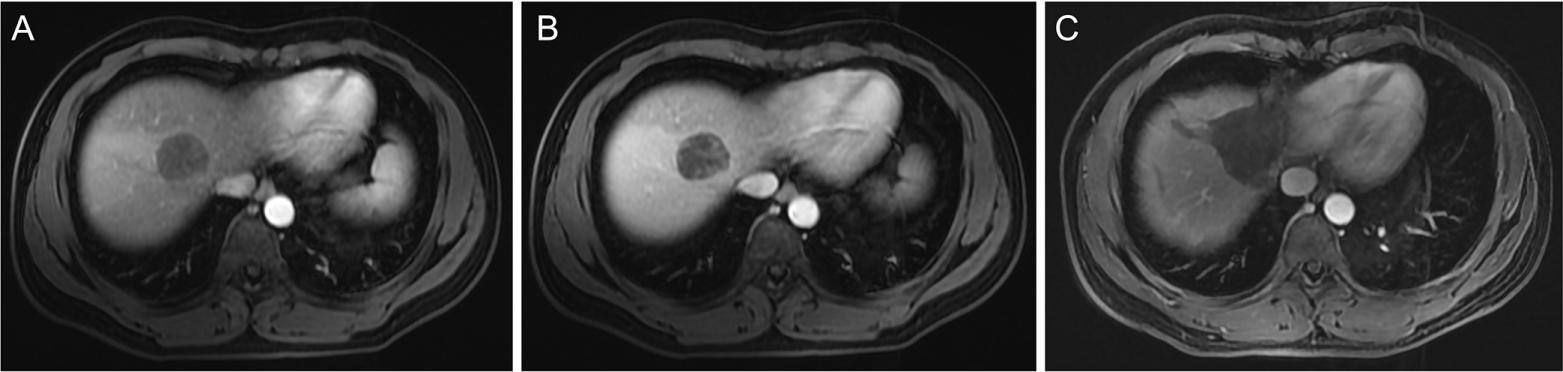
**(A, B)** Enhanced MR at 17 weeks post-bHAIC shows significant regression of the liver lesion following a single session of bHAIC treatment, with complete disappearance of lesion enhancement. **(C)** The patient underwent radical ablation for the liver lesion, and the enhanced MR image at 8 weeks post-ablation revealed that the necrotic area was slightly larger than the original lesion.

**Figure 4:**
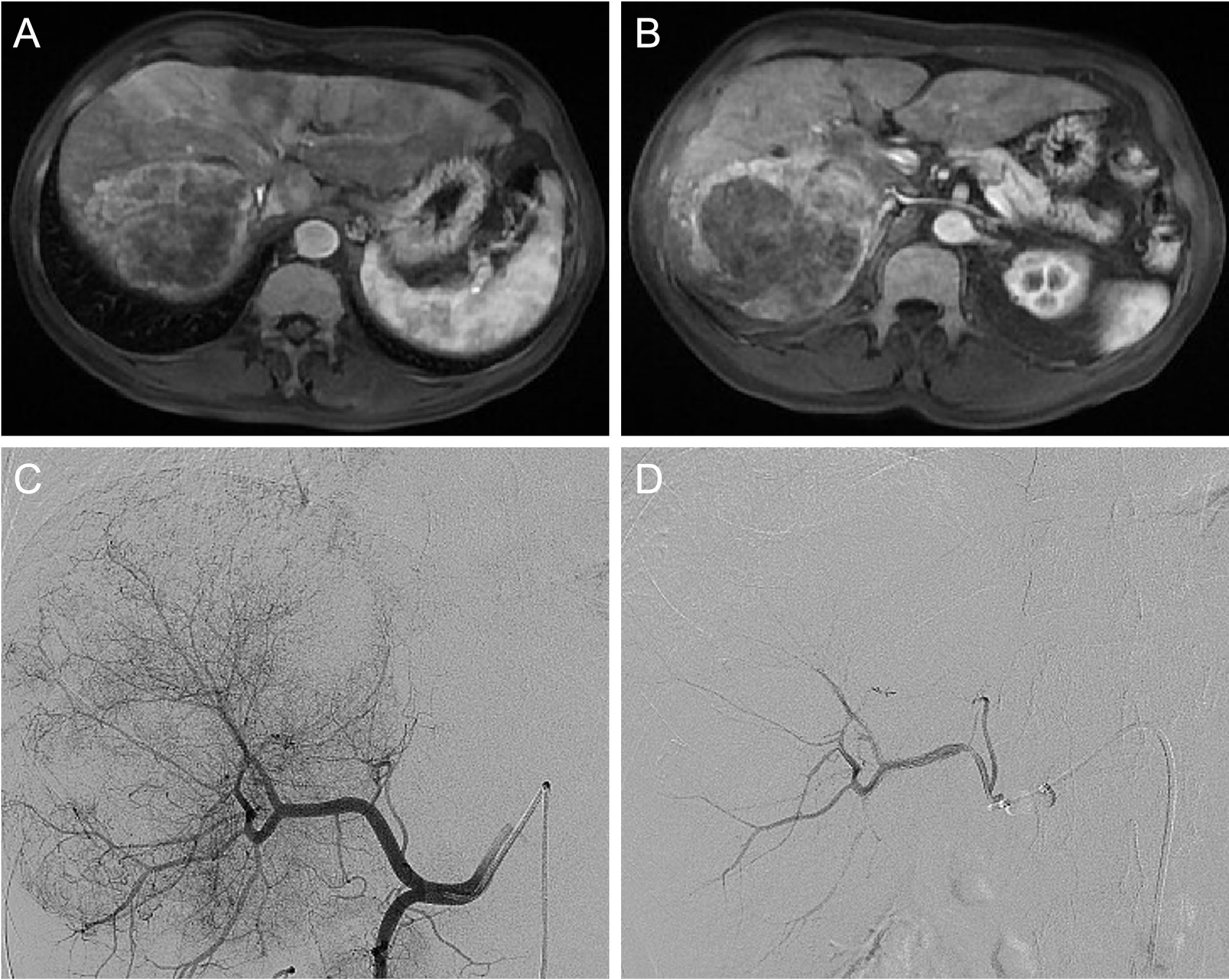
Images in a 67-year-old male with unresectable hepatocellular carcinoma (HCC) who was successfully converted to resectable status after balloon-occluded HAIC (bHAIC) and ultimately underwent radical resection. **(A, B)** Pretreatment enhanced MR shows a 113 × 106 mm lesion in segments VI and VII accompanied by multiple satellite nodules and a Vp3-type portal vein tumor thrombus. **(C, D)** Selective arteriographic images show a giant HCC **(C)** and the position of the balloon catheter occlusion **(D)**.

**Figure 5:**
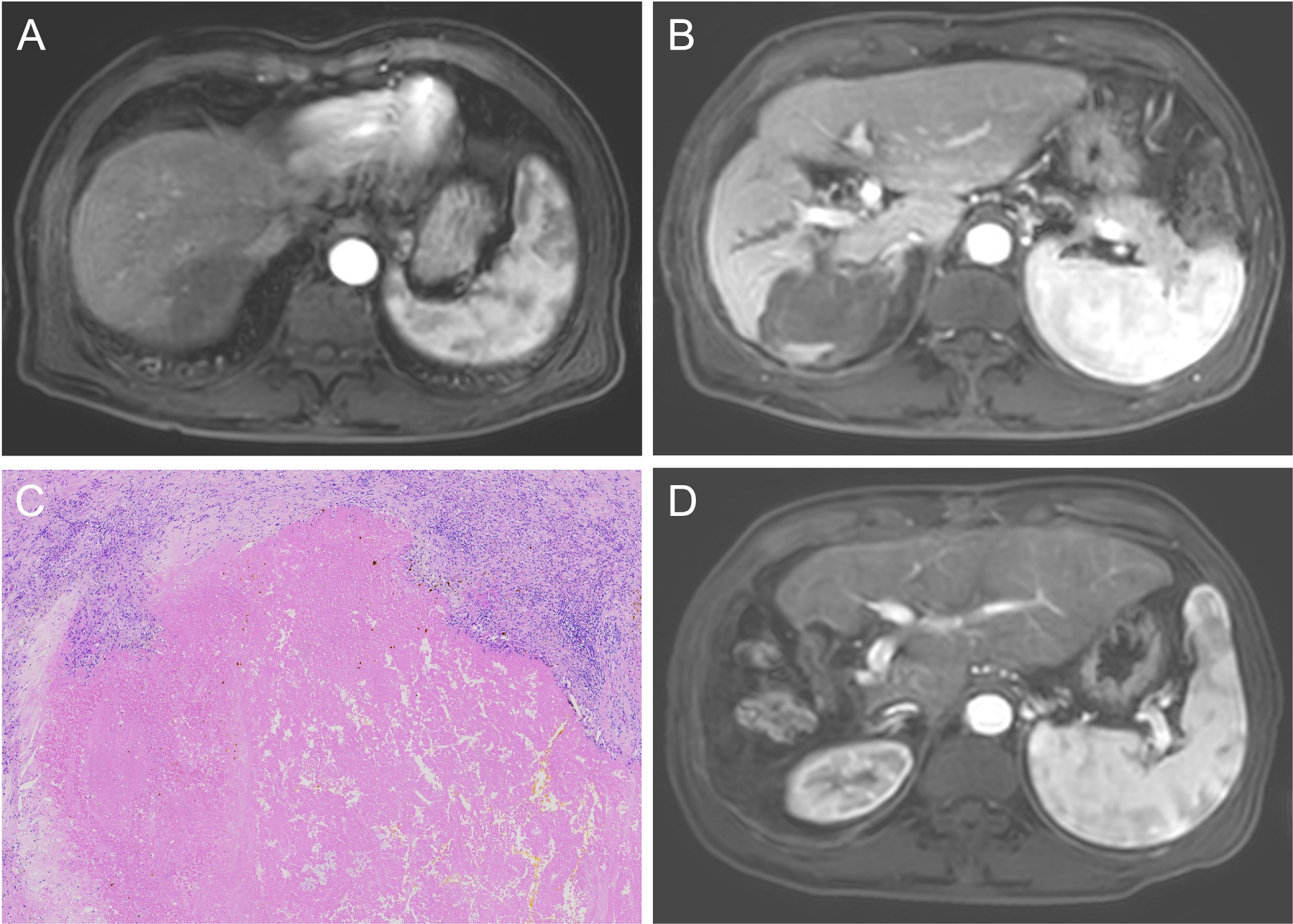
**(A, B)** Enhanced MR image 26 weeks post-bHAIC shows significant regression of the liver lesion, with complete disappearance of lesion enhancement and resolution of the tumor thrombus in the right portal vein branch. **(C)** The patient underwent radical resection. Postoperative pathology showed no viable tumor cells, consistent with complete pathological response. **(D)** Enhanced MR image at 5 weeks post-resection revealed that there was no residual tumor.

From April 2024 to December 2024, a total 53 eligible patients were included at 5 centers in China. Among them, 50 patients (43 males and 7 females; median age, 60.5 years), who had successfully underwent bHAIC treatment, were enrolled in the patients population (**Figure 1**). **Table 1** shows baseline characteristics of the participants (mITT population). The three patients in this study, despite being at the BCLCA stage, were classified as inoperable for the following reasons: Two cases of Child-Pugh B grade.2) Suboptimal tumor location (1 case); 3) Inadequate future residual liver volume (2 cases). Of the participants, 41 (82%) patients were treatment-naive, whereas 9 (18%) had disease progression post-TACE and/or HAIC, with or without concurrent tyrosine kinase inhibitor (TKI) therapy. A majority, 45 (90%), had hepatitis B virus (HBV) etiology and Child-Pugh A liver function, with 37 (74%) patients in Barcelona Clinic Liver Cancer stage C. As of January 23, 2025, a total of 87 bHAIC-FO sessions were completed (median, 2; range, 1–3), with all patients having at least one post-bHAIC follow-up, and 90% having two or more post-bHAIC follow-ups. Furthermore, 13 (26%) patients received subsequent treatments post-bHAIC-FO. Two patients, initially with unresectable HCC, became resectable after bHAIC-FO treatment. One patient underwent radical resection (**Figures 2**, **3**) and the other underwent radical ablation (**Figures 4**, **5**). Of all enrolled patients, 5 (10%) individuals who had previously received TKI therapy continued their original systemic regimen or switched to a second-line drug regimen following bHAIC-FO treatment. Meanwhile, 27 (54%) treatment-naive patients were subsequently treated with lenvatinib monotherapy or combined with immune checkpoint inhibitors. Overall, this interim analysis included 50 patients for safety assessment and 45 patients for efficacy evaluation (**Figure 1**).

**Table 1:** Baseline characteristics in mITT population ( n= 50).

Characteristic	Value
Age*	60.5 (53.0–68.0)
**Sex**	
Female	7 (14)
Male	43 (86)
ECOG performance status	
0–1	40 (80)
2	10 (20)
Etiology	
Hepatitis B	45 (90)
Hepatitis C	2 (4)
Unknown/other	3 (6)
BCLC stage	
A	3 (6)
B	10 (20)
C	37 (74)
Child-Pugh class	
A	45 (90)
B7	5 (10)
Diameter of largest nodule (mm) *	103.9 (67.0–136.3)
Tumor number	
1–3	6 (12)
>3	44 (88)
**Portal vein invasion**	33 (66)
Vp 1–2	7 (14)
Vp 3	16 (32)
Vp 4	10 (20)
Venous invasion (hepatic vein and/or inferior vena cava)	8 (16)
Macrovascular invasion (portal vein and/or venous invasion)	37 (74)
AFP level (IU/mL)*	951.7 (21.3–19369.0)
Within the normal range	6 (12)
Exceeding the normal range	44 (88)
**Previous therapy received†**	9 (18)
Transarterial chemoembolization	9 (18)
Hepatic arterial infusion chemotherapy	1 (2)
TKI therapy	5 (10)

Unless otherwise specified, categorical data are presented as numbers of participants, with percentages in parentheses.

*Continuous data are presented as medians, with IQRs in parentheses.

†Some patients had more than one previous treatment.

AFP, a-fetoprotein; BCLC, Barcelona Clinic Liver Cancer; ECOG, European Cooperative Oncology Group; mITT, modified Intent-to-Treat; TKI, Tyrosine Kinase Inhibitor.

### Efficacy

Among the 45 evaluable patients who underwent two or more post-bHAIC follow-ups, the best ORR achieved was 53.3% according to RECIST 1.1 criteria and 82.2% according to mRECIST criteria (**Table 2**). Of these patients, 36 were treatment-naive at enrollment, achieving a best ORR of 88.9% by mRECIST. In contrast, the best ORR for the nine patients with a history of prior TACE or HAIC treatments was 55.6%.

**Table 2:** Tumor responses for evaluable patients (n = 45).

	RECIST 1.1			mRECIST		
Variable	Previous treatment received n=9	Treatment naive n=36	Total n=45	Previous treatment received n=9	Treatment naive n=36	Total n=45
Best response						
Complete response	0 (0)	0 (0)	0 (0)	1 (11.1)	14 (38.9)	15 (33.3)
Partial response	4 (44.4)	20 (55.6)	24 (53.3)	4 (44.4)	18 (50.0)	22 (48.9)
Stable disease	5 (55.6)	15 (41.7)	20 (44.4)	4 (44.4)	2 (5.6)	6 (13.3)
Progressive disease	0 (0)	1 (2.8)	1 (2.2)	0 (0)	2 (5.6)	2 (4.4)
Objective response rate	4 (44.4)	20 (55.6)	24 (53.3)	5 (55.6)	32 (88.9)	37 (82.2)
Disease control rate	9 (100)	35 (97.2)	44 (97.8)	9 (0)	34 (94.4)	43 (95.6)

Data are expressed as numbers of patients, with percentages in parentheses.

RECIST 1.1, Response Evaluation Criteria in Solid Tumors version 1.1; mRECIST, Modified RECIST.

Out of the 45 evaluable patients, 15 achieved a complete radiological response, including seven patients whose AFP levels fully normalized (images data are provided in the **Supplementary Material 3**). A total of two patients were diagnosed with PD. Notably, one patient initially classified as having a partial response (PR) was reclassified as PD in subsequent confirmation assessments due to the identification of thoracic vertebral metastases, despite the complete disappearance of intrahepatic lesion enhancement.

**Figure 6:**
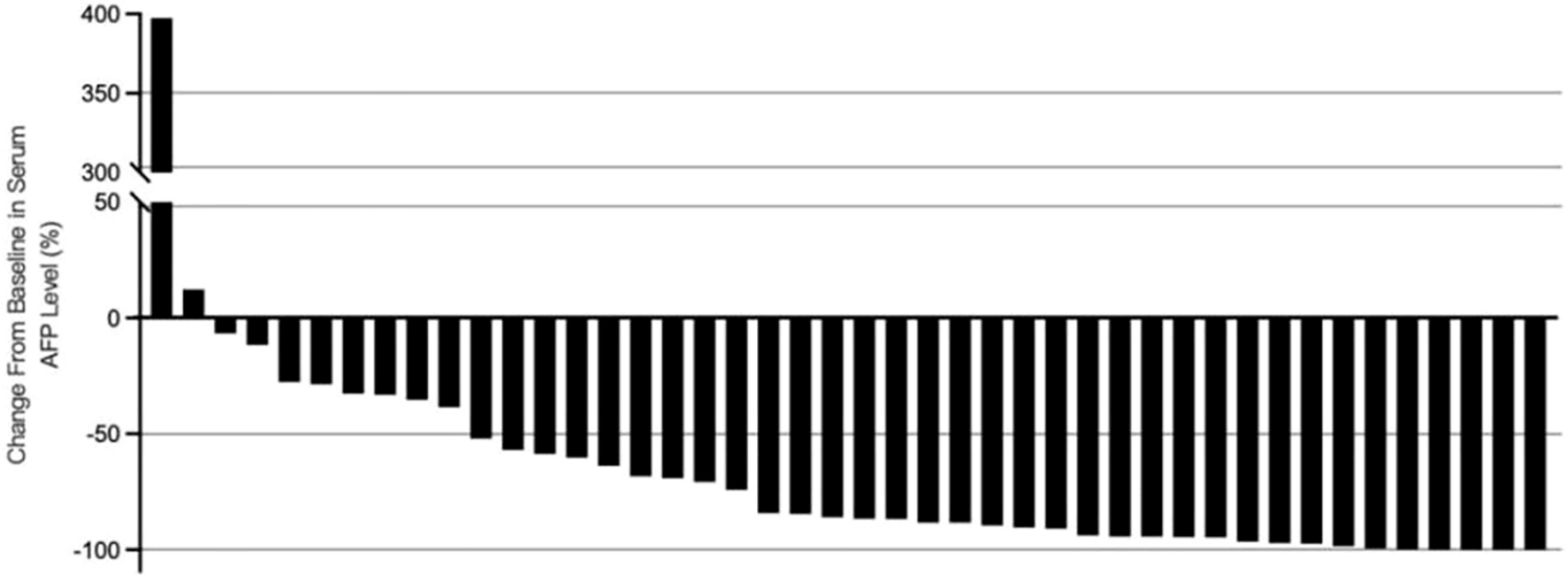
Waterfall plots depicting the maximum change in serum alpha-fetoprotein (AFP) levels for each patient with abnormally elevated AFP levels (n = 44).

Among the 44 patients with abnormally elevated AFP levels, 40 (90.9%) experienced a reduction of AFP of over 20% after bHAIC-FO treatment, with 34 (77.3%) achieving a reduction exceeding 50%. Waterfall plots show the maximum changes in AFP levels for each patient (**Figure 6**).

### Safety

We analyzed the safety of the bHAIC - FO treatment in all 50 patients who underwent at least one follow - up after the procedure. Importantly, no treatment-related deaths reported. The most frequently observed AEs included abdominal pain during arterial infusion and abnormal hepatic function, as detailed in **Table 3**.

**Table 3:** Adverse events attributable to bHAIC-FO treatment with greater than 5% frequency in mITT Population (n = 50).

Adverse events	Total	Grade 1–2	Grade 3–4
Abdominal pain	41 (82)	13 (26)	28 (56)
Abnormal hepatic function			
Increased AST/ALT	45 (90)	22 (44)	23 (46)
Hyperbilirubinemia	18 (36)	18 (36)	0
Hypoalbuminemia	7 (14)	7 (14)	0
Anemia	3 (6)	3 (6)	0
Leukocytosis	5 (10)	5 (10)	0
Increased serum creatinine	5 (10)	5 (10)	0
Nausea/vomiting	20 (40)	20 (40)	0
Fever	21 (42)	21 (42)	0
Arterial injury	5 (10)	5 (10)	0

Data are expressed as numbers of patients, with percentages in parentheses.

ALT, alanine transaminase; AST, aspartate transaminase; bHAIC-FO, balloon-occluded hepatic arterial infusion chemotherapy based on FOLFOX regimen.

Specifically, 23 patients experienced Grade 3–4 increases in aspartate aminotransferase and/or alanine aminotransferase within 1–3 days post-bHAIC. Additionally, 18 patients developed Grade 1–2 hyperbilirubinemia. Remarkably, most of these biochemical parameters returned to baseline levels within a week of bHAIC-FO treatment without any special intervention needed.

Two patients with large tumor burdens, exceeding 50% of liver volume, experienced aspartate aminotransferase levels rising more than 20 times the ULN within 12 hours post-bHAIC. Abdominal CT showed marked hypodensity changes in the tumor area compared with baseline, suggesting the occurrence of tumor lysis syndrome. As a preventive measure, continuous renal replacement therapy was administered for an additional 24 to 48 hours to prevent renal dysfunction and electrolyte imbalances, with further clinical data available in **Supplementary Material 4**.

In addition, 21 patients with high tumor burden exhibited postoperative fever and elevated neutrophil ratio, necessitating intravenous broad-spectrum antibiotics. Moreover, five patients demonstrated varying degrees of stenosis and/or dilation in the hepatic artery branches at the balloon occlusion site during subsequent interventional treatment, although they remained asymptomatic.

Importantly, compared with baseline, patients did not exhibit a noticeable decrease in white blood cells and platelet counts post-bHAIC, and no patients discontinued treatment due to bHAIC-related complications.

## Discussion

FOLFOX-based systemic chemotherapy is effective for HCC (14), but not optimal. Switching to HAIC enhances the antineoplastic potency of the FOLFOX, yet some HCC patients remain unresponsive or show disease progression (9). In this phase II study, a divergent approach was adopted to explore strategies aimed at optimizing the efficacy of HAIC.

HAIC efficacy depends on the local concentration and retention time of chemotherapeutic drugs (17). Compared with HAIC, bHAIC is hypothesized to confer two key advantages. Firstly, balloon occlusion of the hepatic artery or its major branches reduces the occluded vessel blood flow, owing to limited intrahepatic collateral circulation (18, 19), minimizing drug dilution and enhancing the local plasma drug concentration in the tumor-feeding artery. Secondly, the decreased residual blood flow velocity post-occlusion, as indicated by delayed contrast clearance (20, 21), may prolong the intrahepatic drug passage time, increasing tumor cell exposure to the chemotherapeutic drugs. Furthermore, the notable disparity in blood supply between normal liver parenchyma, which derives 75%–80% of its blood from the portal vein (22, 23), and HCC, which is artery-dependent (24), establishes a pressure gradient following occlusion. This gradient directs the drugs toward the tumor’s vascular compartment while sparing normal hepatocytes, optimizing the therapeutic window of bHAIC (21).

According to mRECIST, among 45 evaluable patients, ORR was 55.6% for those previously treated with TACE or HAIC, while it reached up to 88.9% in treatment-naive patients, suggesting that bHAIC may offer improved therapeutic prospects for treatment-naive HCC. Two key factors were identified to analyze the potential reasons underlying the stark discrepancy in tumor ORR: firstly, as noted, repeated TACE may reduce post-occlusion blood flow velocity insufficiently, potentially diminishing the bHAIC efficacy. Secondly, multiple TACE or HAIC sessions might reduce the tumor chemotherapy sensitivity, thereby impacting outcomes. The ORR observed in our study is higher than those reported with HAIC (25, 26). The literature indicates that the overall ORR for patients with advanced HCC undergoing traditional HAIC treatment is 27.3%. This rate is 30.5% for patients classified as Child-Pugh grade 5/6, 28.2% for grade 7, and only 13.8% for grades 8/9. This study demonstrated that the ORR of b-HAIC treatment was 82.2% based on mRECIST criteria, with a noTable 88.9% for newly diagnosed patients. This rate significantly exceeds the response rates observed in each subgroup of traditional HAIC reported in existing literature.

The safety profile of this trial was composed of AEs related to chemotherapy drugs and bHAIC. Like HAIC, gastrointestinal toxicity and biliary sclerosis are also noteworthly concerns in bHAIC (27). During HAIC, chemotherapy drugs enter the stomach and/or duodenum through hepatic artery branches, causing ectopic perfusion and mucosal inflammation or ulcers in the affected organs. To prevent these occurrences, HAIC requires hepatic artery skeletonization to embolize gastric/intestinal feeders; yet, incomplete smaller vessels embolization and anatomical variations limit ectopic perfusion prevention (28, 29). Biliary sclerosis also results from ectopic perfusion, though bile duct arterial blood supply is comparatively more complex (30). Bile ducts possess a surface peribiliary vascular plexus (PVP) (31, 32); during HAIC, drugs enter the PVP via the hepatic and cystic arteries, injuring bile duct. However, in cases of hepatic artery occlusion, the PVP serves as a connection between the hepatic and gastroduodenal arteries, which in turn provides compensatory collateral arterial blood supply to the liver via the descending marginal artery (31–33). Therefore, drugs administered via bHAIC may not enter the PVP, protecting the bile ducts. Similarly, bHAIC may protect gastrointestinal vessels through a comparable mechanism where hepatic artery balloon occlusion forms collateral circulation to provide compensatory collateral blood flow for the proximally obstructed hepatic artery (18, 34). The absence of gastric and bile duct-related complications in our study supports this hypothesis.

Hepatic artery balloon occlusion alters hepatic hemodynamics, affecting chemotherapeutic drugs action administered via infusion. Hence, drug dosages used in conventional HAIC should be adjusted for bHAIC. In this study 110 mg/m^2^ of oxaliplatin and 1100 mg/m^2^ of fluorouracil were used to balance efficacy and safety. Although various HAIC regimens exist beyond FOLFOX, the most effective one is yet to be determined (17). Given the potential variability in chemosensitivity among HCC patients, the combination of multiple drugs may enhance efficacy, but raises concerns regarding systemic and hepatic toxicity. bHAIC addresses these concerns by promoting the drug accumulation and prolonging retention time in tumor while minimizing normal tissue impact, achieving reduced toxicity and enhanced efficacy. In this study, the doses of oxaliplatin and fluorouracil doses were lower than those used in HAIC-FO, with the fluorouracil at just 39% of the conventional level (9, 11). The reduction in the total amount of drugs decreased toxicity and opens up an opportunity for future exploration of combinatorial chemotherapies.

This study has several limitations. First, 90% of participants had HBV-related HCC, so the efficacy and safety of bHAIC-FO in non-HBV HCC require further validation. Second, this research is an interim analysis that was predetermined. In accordance with the study plans, after all patients completed enrollment and reached the designated follow-up period, we will provide the pertinent survival statistics. Quantitative occlusion parameters (e.g., stump pressure, contrast clearance time, exact coverage fraction) were not systematically captured in this interim analysis, limiting procedural reproducibility assessment. In addition, the mRECIST in this study underwent independent evaluation by senior radiologists at each center. Due to resource limitations, a central review was not performed, potentially introducing bias. Future research should include an independent imaging review mechanism to improve the reliability of the findings. The proposed advantages of bHAIC-enhanced local drug concentration and prolonged drug retention time remain theoretical assumptions. The underlying mechanisms linking bHAIC require confirmation. Further verification is needed in clinical settings or through basic research using microfluidic chips. Additionally, a systematic long-term imaging follow-up to assess balloon-related vascular injuries was not performed. In future studies, balloon-related vascular complications should be considered an independent safety endpoint, necessitating regular imaging monitoring. Finally, because the best ORR was evaluated within 4 weeks after bHAIC and subsequent treatments typically take over a month to work, their effect on ORR is minimal. However, PFS and OS could be strongly influenced by these later therapies, so the survival benefit should not be attributed solely to bHAIC−FO.

In conclusion, bHAIC-FO demonstrates high ORR and acceptable safety in unresectable HCC, particularly in advanced portal vein invasion patients. We anticipated that the observed high ORR may translate into survival benefits, which will be confirmed in the final analysis.

## Data Availability

The original contributions presented in the study are included in the article/**Supplementary Material**. Further inquiries can be directed to the corresponding author.
