# Supplementary material for: Balloon-occluded hepatic arterial infusion for unresectable hepatocellular carcinoma: a phase II trial interim analysis
Source: Front Oncol. 2026 Jun 1;16:1761615. doi: 10.3389/fonc.2026.1761615 (PMC13265302; doi:10.3389/fonc.2026.1761615)
Supplement: Supplementary file 1 [file DataSheet1.docx]

Supplemental Material

Balloon-occluded hepatic arterial infusion for unresectable hepatocellular carcinoma: a phase II trial interim analysis

Supplemental Material 1

**Study Protocol**

**Patients**
This prospective, single-arm, multicenter study plans to enroll patients from 10 research centers. Patients were eligible for inclusion if they met the following criteria: (a) age 18 years or older; (b) diagnosis of hepatocellular carcinoma according to the AASLD guidelines; (c) presenting with unresectable disease (including advanced HCC, unsuitable for TACE, or progression after two TACE sessions), without extrahepatic metastasis, and with obvious arterial phase enhancement of intrahepatic lesions; (d) Child-Pugh class A or B (score of 7); (e) Hemoglobin (HB) ≥90 g/L; White Blood Cell count (WBC) ≥3.0×10^9/L; Platelet count (PLT) ≥50×10^9/L; AST and ALT (transaminases) ≤5.0 × ULN; Total Bilirubin (T-BIL) ≤1.5 × ULN; Creatinine (CRE) ≤1.5 × ULN; (f) Eastern Cooperative Oncology Group (ECOG) performance status score of 0-2; and (g) life expectancy exceeding 3 months. Key exclusion criteria were as follows: (a) portal vein tumour thrombus in the main trunk simultaneously involving bilateral first-order branches without adequate collateral vessels; (b) Child-Pugh score ≥8; (c) organ (cardiac, renal) insufficiency rendering intolerance to HAIC; (d) definite extrahepatic metastasis, history of other malignancies potentially impacting current HCC treatment, or active infection requiring systemic treatment (excluding HCV or HBV); (e) previous hepatobiliary surgery altering vascular anatomy; (f) prior treatment with immune checkpoint inhibitors (PD-1 or PD-L1) for over 3 months, or recipients of allogeneic tissue/solid organ transplants; (g) current pregnancy or lactation; and (h) concurrent or prior participation in interventional clinical trials potentially interfering with results, or investigator judgment of non-beneficial participation.

The sample size was calculated using PASS software. With a one-sided significance level (α) of 0.05, power (1-β) of 0.8, a assumed baseline objective response rate (P0) of 0.3, an expected rate (P1) of 0.5, and a 10% dropout rate, the study plans to enroll a total of 49 eligible hepatocellular carcinoma patients. Consecutive enrollment will be used for this single-arm cohort study.

Combination with targeted or immunotherapy is not prohibited.

Study Approval
The study was approved by the Ethics Committee of The First Affiliated Hospital of Dalian Medical University. All participants provided written informed consent before any study-related procedures were performed.

Data Collection
Demographic data, medical history, vital signs, clinical symptoms and signs, treatment history, laboratory results (complete blood count, biochemistry, tumor markers AFP, urinalysis, stool routine), imaging data, and ECOG scores were collected (details provided in the study protocol). All data were collected prospectively.

Treatment Details
The b-HAIC protocol was as follows: Oxaliplatin 110mg/m² (intra-arterial infusion over 2 hours), Leucovorin (lv) 300mg/m² (intravenous infusion over 2 hours), followed by 5-Fluorouracil (5-Fu) 1100mg/m² (continuous intra-arterial infusion over 24 hours). The procedure involved percutaneous hepatic artery catheterization, placement of a balloon catheter (in the proper hepatic artery, left/right hepatic artery level, or common hepatic artery after blocking the gastroduodenal artery), balloon inflation to an appropriate diameter based on the target artery, catheter fixation, and subsequent 24-hour chemoperfusion via the indwelling sheath.

Patients received the first b-HAIC cycle at week 0. Subsequent cycles were administered every 4 weeks based on response evaluation, for a maximum of 3 cycles. Response was assessed radiologically (using RECIST 1.1 or mRECIST as specified) 4 weeks after each cycle. Patients with Complete Response (CR) stopped b-HAIC and entered follow-up (every 8 weeks until progression, upon which original b-HAIC could be reinitiated). Those with Partial Response (PR) or Stable Disease (SD) continued b-HAIC cycles (Q4W). Patients with Progressive Disease (PD) exited the study for alternative therapy.

Outcome Variables and Follow-up
The primary efficacy endpoint was Objective Response Rate (ORR). Secondary efficacy endpoints included Alpha-fetoprotein (AFP) response, Disease Control Rate (DCR), Duration of Response (DOR), Progression-Free Survival (PFS), Overall Survival (OS), and incidence of Adverse Events (AEs).

Follow-up was scheduled at 4, 8, 16, 24, 32, 40, 48, 56, and 64 weeks post-initially, including imaging (contrast-enhanced CT/MR of the upper abdomen), laboratory tests (CBC, biochemistry, AFP), assessment of AEs, and ECOG score. Follow-up continued for at least 1 year. The frequency was Q4W during active b-HAIC treatment, switching to Q8W after achieving CR, completing 3 cycles, or if treatment was discontinued due to intolerance despite response (PR/SD). Telephone follow-ups (survival status, performance status, key lab results) were conducted if hospital visits were impossible. Patients who withdrew were followed per protocol until study end where possible. A pre-planned interim analysis was triggered when approximately 50% of the target sample size had been enrolled and all enrolled patients had completed at least 3 months of follow‑up. The interim analysis was designed to evaluate both efficacy (objective response rate) and safety. The analysis and the decision to release the interim data were reviewed and approved by the hospital's Data Safety Monitoring Board. No formal early stopping rules for futility or superiority were predefined.

Safety Assessment
Safety was assessed based on patient interviews (including direct questioning about AEs/concomitant medications), physical examinations, vital signs, weight, ECG, laboratory safety parameters (e.g., CBC, serum creatinine, BUN, electrolytes, liver enzymes), and the recording and assessment of all AEs and SAEs, including their severity (mild, moderate, severe) and relationship to study treatment, according to standard definitions (CTCAE). All AEs, regardless of relationship, were followed until resolution, return to baseline, or stabilization.

Subject Discontinuation (Withdrawal)
Subjects could withdraw at any time. Investigators could withdraw subjects for safety reasons (e.g., SAEs, comorbidities), poor compliance, or treatment failure (progression/death). Procedures for handling withdrawn subjects, including reason documentation, efforts to complete final assessments, and AE follow-up, were detailed in the protocol.

Statistical Analysis
Statistical analysis will be performed on the Full Analysis Set (FAS) and/or Per Protocol Set (PPS). Descriptive statistics will be used for demographics and baseline characteristics. Efficacy analyses will employ appropriate methods (e.g., logistic regression for response rates, Kaplan-Meier for survival endpoints). Safety data will be summarized descriptively. The statistical significance level (α) is set at 0.05, two-sided. Specific statistical methods for primary and secondary endpoints were pre-defined in the protocol. Propensity score matching or inverse probability weighting was not planned due to the single-arm design.

Supplemental Material 2

bHAIC procedure

The procedures were performed via femoral artery access or radial artery access. Digital Subtraction Angiography was carried out to assess the anatomy of the hepatic artery, tumor vascularization map, and lesion’s feeder vessels. The optimal occlusion position of the balloon catheter and the selection of the balloon diameter were determined based on the angiography information. For example, if both the left and right hepatic arteries were involved in tumor blood supply, the optimal location of the balloon catheter was the proper hepatic artery. However, if lesions were fed or predominantly fed by one artery (left hepatic artery or right hepatic artery), the specific artery would be selected as the best location for balloon catheter placement, while other feeding branches could be treated with precision transarterial chemoembolization. If occlusion at the proper hepatic artery could not be achieved due to vascular anatomical factors, complete embolization of the gastroduodenal artery was performed first, followed by occlusion at the level of the common hepatic artery. During hepatic artery occlusion, the balloon was inflated to a diameter that matches the size of the vessel in which it was located. Balloon catheters were available in 2.4F (Caidiolink Science, China), 4F, and 5.5F sizes (Hengrui Medicine, China), with various balloon diameters when dilated. Typically, for occlusion at the level of the proper hepatic artery or common hepatic artery, the balloon was inflated to a diameter of around 5–8 mm, while for occlusion at the level of the left or right hepatic artery, the balloon diameter was typically around 3–5 mm. A modified FOLFOX regimen, which included oxaliplatin (110 mg/m^2^ arterial infusion over two hours), leucovorin (300 mg/m^2^ intravenously over two hours), and fluorouracil (1100 mg/m^2^ continuous arterial infusion over 24 hours), was adopted for bHAIC. Prior to intra-arterial chemotherapy infusion, 5 mg of dexamethasone was routinely administered via balloon catheter to mitigate vascular irritation, while a subcutaneous injection of 5–10 mg of morphine was given for analgesia. For episodic abdominal pain during infusion, 5 mL of 0.5% lidocaine can be delivered through the balloon catheter for pain relief. After each bHAIC procedure, the balloon catheter and sheath were removed. Repetitive catheterization was performed in the next bHAIC procedure.

**bHAIC underlying technical principles**

Balloon-occluded hepatic arterial infusion (bHAIC) exploits the liver’s dual blood supply to amplify intratumoral chemotherapy while sparing normal parenchyma. Inflating a micro-balloon within the proper or segmental hepatic artery abolishes antegrade flow; because intra- and extra-hepatic collaterals are limited, residual arterial pressure and blood velocity fall by 30–70% . Angiography shows delayed contrast wash-out, signalling a doubling of the intra-hepatic first-pass time for oxaliplatin and 5-FU and a two- to four-fold rise in peak intratumoral drug concentration compared with conventional HAIC. HCC nodules, which depend almost exclusively on arterial input, experience an about 90 % drop in vascular pressure, whereas surrounding hepatocytes—perfused predominantly by the portal vein—maintain sinusoidal pressure. This pressure gradient steers the infused agents into the low-pressure tumor vascular space and limits entry into normal sinusoids, achieving “haemodynamic dose intensification” without higher systemic exposure. Consequently, protocol doses could be reduced (oxaliplatin 110 mg m⁻², 5-FU 1100 mg m⁻²) yet still produce high response rates and no grade ≥3 myelosuppression. Deeper balloon positions that minimize collateral vessels correlate with lower stump pressures and higher objective responses, whereas prior multiple TACE sessions may enlarge collaterals and blunt the effect, underscoring the importance of patient selection and optimized occlusion strategy.

Supplemental Material 3

Fifteen cases of completed response (CR) imaging data with legends attached

Case 1

A 69-year-old male with a history of hepatitis B was diagnosed with unresectable hepatocellular carcinoma (HCC) in segments V and VIII, accompanied by Vp2-type portal vein thrombus in the right branch and hepatic vein tumor thrombus, with an alpha-fetoprotein (AFP) level at 38,321 IU/mL (reference range 0–5.8 IU/mL). (A–D) Pretreatment contrast-enhanced Computed Tomography (CT) showed multiple intrahepatic lesions with significant enhancement and invasion of the right branch of the portal vein. (E–H) After two sessions of balloon-occluded hepatic arterial infusion chemotherapy (bHAIC) based on a FOLFOX regimen (bHAIC-FO), the contrast-enhanced CT upon re-examination showed a significant reduction in the lesions and disappearance of tumor enhancement. According to the mRECIST criteria, the therapeutic effect was assessed as CR.

| 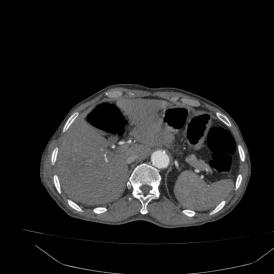 | 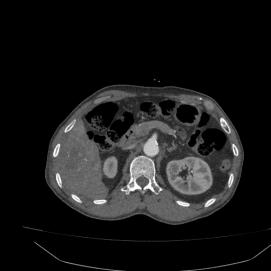 |
| --- | --- |
| A | B |
| 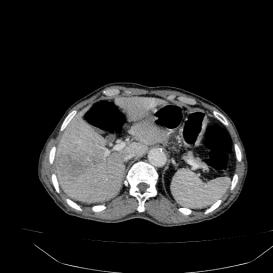 | 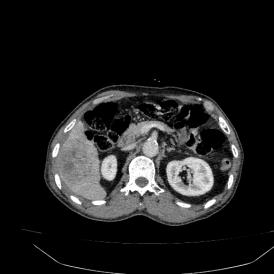 |
| C | D |
| 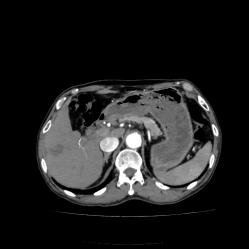 | 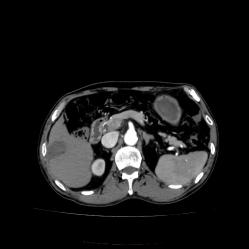 |
| E | F |
| 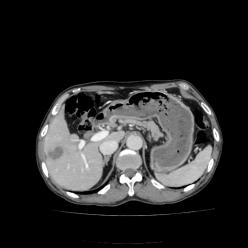 | 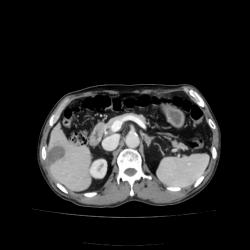 |
| G | H |
| Supplemental Figure 1. Contrast-enhanced CT images of Case 1 at different stages. | |

Case 2

A 69-year-old female with a history of hepatitis B, diagnosed with HCC for two years, had undergone multiple sessions of transarterial chemoembolization (TACE) treatment. She has multiple scattered HCC lesions in the left, right, and caudate lobes of the liver, accompanied by a Vp3-type tumor thrombus in the left branch of the portal vein, with AFP level at 447.7 IU/mL (reference range 0–5.8 IU/mL). (A, B) Pretreatment contrast-enhanced Magnetic Resonance (MR) revealed multiple HCC lesions in segments I, IV, VII, and VIII, with significant arterial phase enhancement. (C, D) Post-bHAIC follow-up examinations revealed early enhancement of the portal vein in the arterial phase, indicating the presence of an arterioportal shunt. The enhancement in multiple HCC lesions and the thrombus in the left branch of the portal vein disappeared. According to the mRECIST criteria, the therapeutic effect was assessed as CR.

| 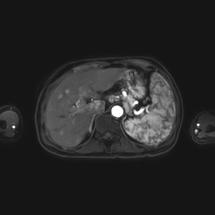 | 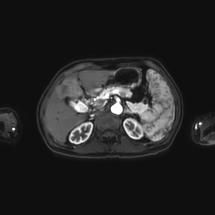 |
| --- | --- |
| A | B |
| 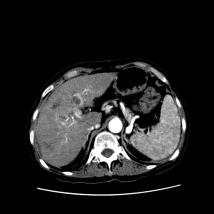 | 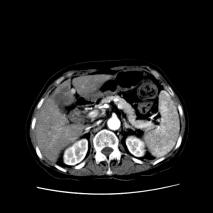 |
| C | D |
| Supplemental Figure 2. Contrast-enhanced CT images of Case 2 at different stages. | |

Case 3

See Case 1 in Figure 2 of the main text.

Case 4

A 69-year-old female with a history of hepatitis B was diagnosed with a massive HCC in the left hepatic lobe, accompanied by Vp4-type portal vein tumor thrombus. AFP was within the normal range. (A–D) Pretreatment contrast-enhanced CT showed massive HCC lesions in segments II, III, and IV with tumor thrombus in the left and right branches of the portal vein and the main trunk. (E–H) After two sessions of bHAIC-FO, re-examination with contrast-enhanced MR revealed a significant reduction in lesion size, along with the disappearance of tumor and portal vein thrombus enhancement. According to the mRECIST criteria, the therapeutic effect was assessed as CR.

| 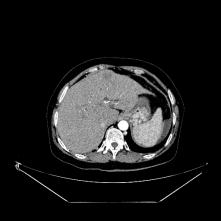 | 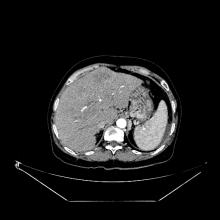 | 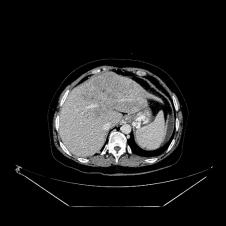 | 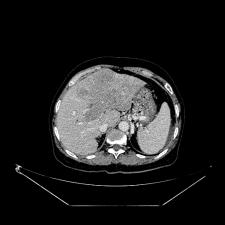 |
| --- | --- | --- | --- |
| A | B | C | D |
| 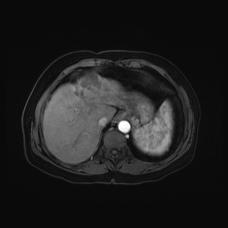 | 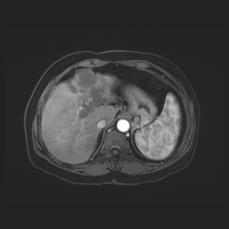 | 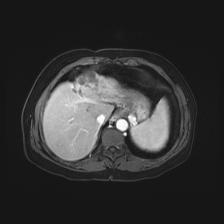 | 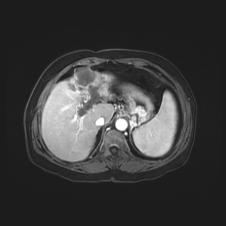 |
| E | F | G | H |
| Supplemental Figure 3. Contrast-enhanced CT images of Case 4 at different stages. | | | |

Case 5

See Case 2 in Figure 2 of the main text.

Case 6

A 45-year-old male with a history of hepatitis B was diagnosed with a massive HCC in the right hepatic lobe, accompanied by Vp4-type portal vein tumor thrombus, with AFP level at 8.6 IU/mL (reference range 0–5.8 IU/mL). (A–D) Pretreatment contrast-enhanced MR showed HCC lesions in segments V, VI, VII, and VIII with tumor thrombus in the left and right branches of the portal vein. (E–H) The patient underwent only one bHAIC-FO treatment, and follow-up post-bHAIC showed a significant reduction in the lesions, disappearance of tumor enhancement, and regression of the left branch portal vein tumor thrombus. According to the mRECIST criteria, the therapeutic effect was assessed as CR.

| 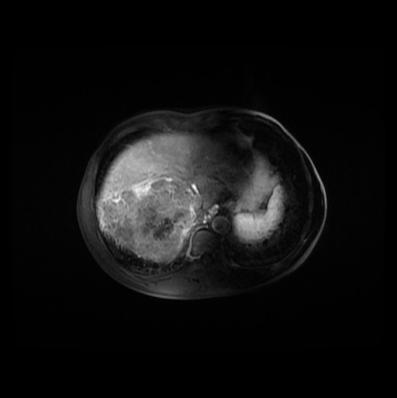 | 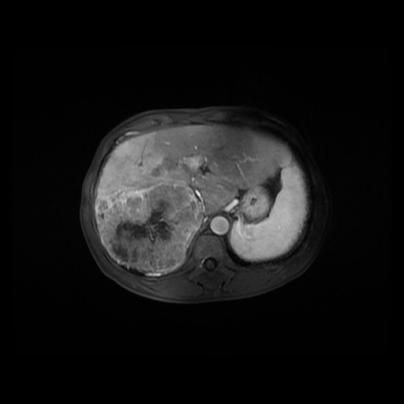 | 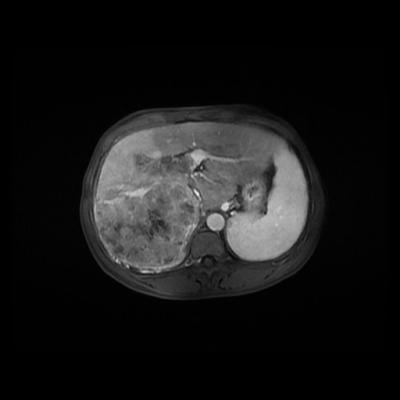 | 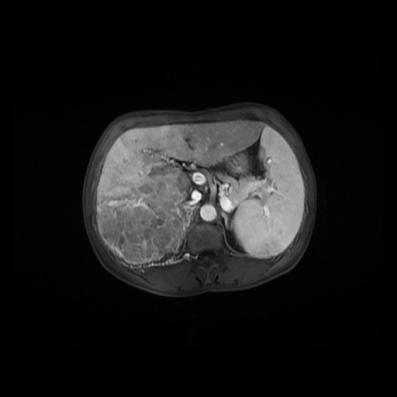 |
| --- | --- | --- | --- |
| A | B | C | D |
| 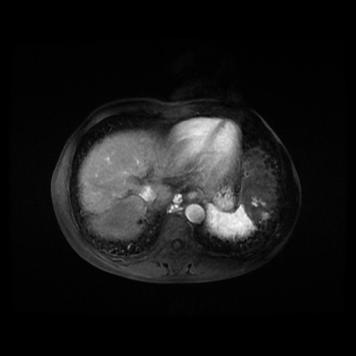 | 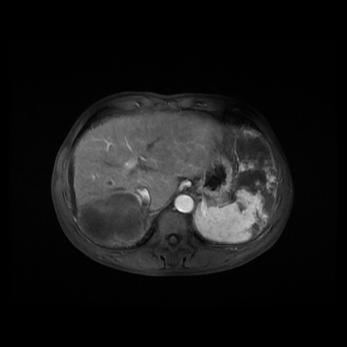 | 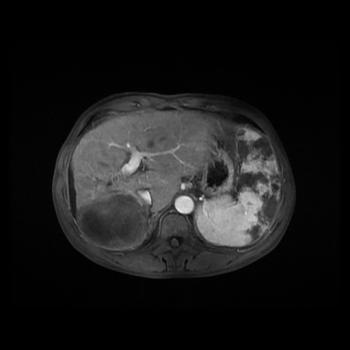 | 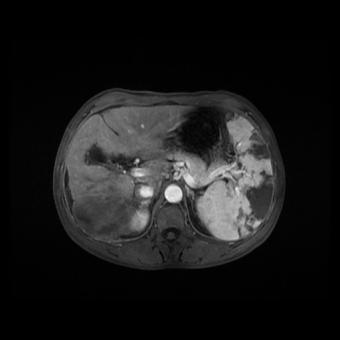 |
| E | F | G | H |
| Supplemental Figure 4. Contrast-enhanced CT images of Case 6 at different stages. | | | |

Case 7

A 49-year-old male with a history of hepatitis B was diagnosed with a massive HCC in the left hepatic lobe, accompanied by Vp4-type portal vein tumor thrombus, with AFP level at 854 IU/mL (reference range 0–5.8 IU/mL). (A–C) Pretreatment contrast-enhanced MR showed a massive HCC lesion in segments II, III, and IV, with tumor thrombus in the left branches of the portal vein and the main trunk. (D–F) After two sessions of bHAIC-FO, re-examination with contrast-enhanced MR showed a significant reduction in the lesion size, the disappearance of tumor enhancement, and regression of the main trunk of the portal vein thrombus. According to the mRECIST criteria, the therapeutic effect was assessed as CR.

| 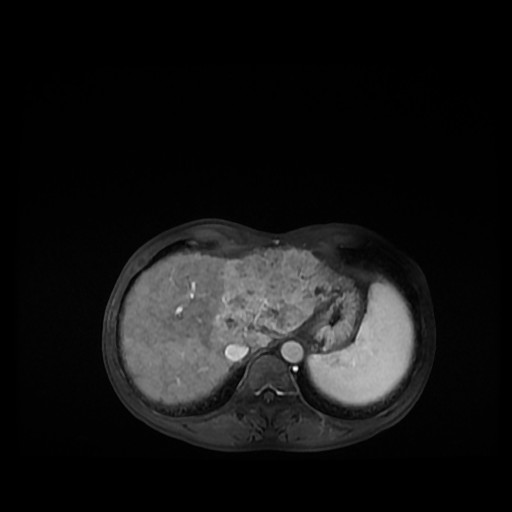 | 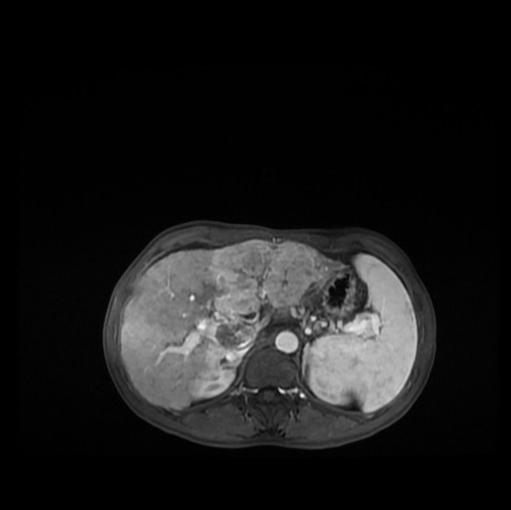 | 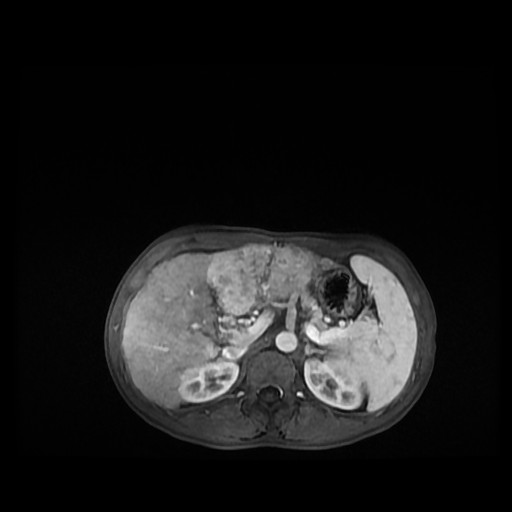 |
| --- | --- | --- |
| A | B | C |
| 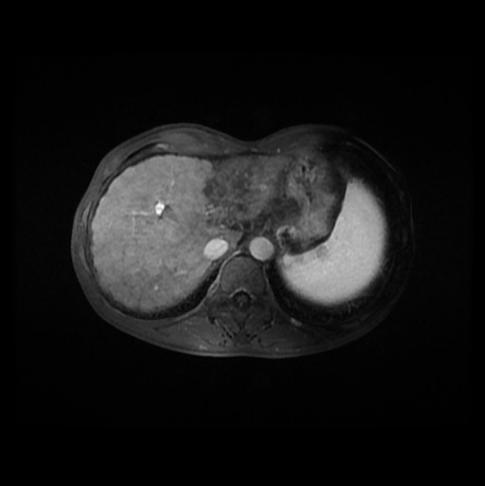 | 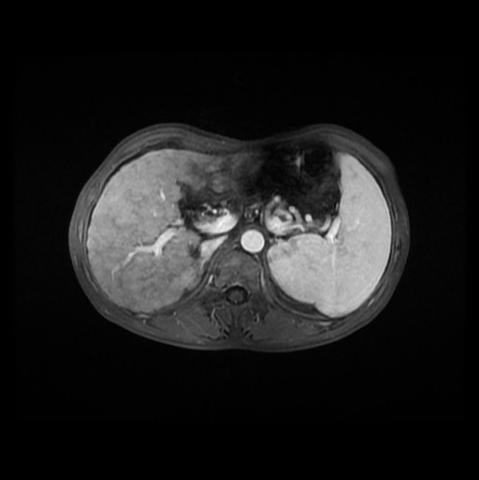 | 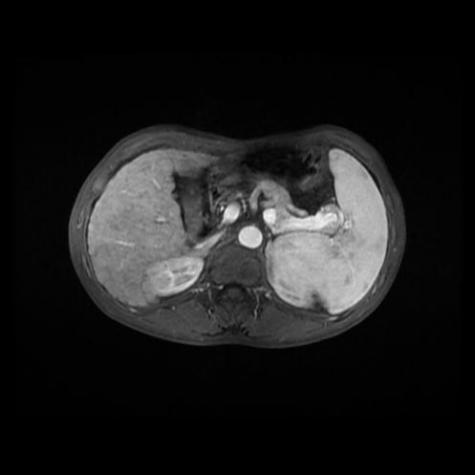 |
| D | E | F |
| Supplemental Figure 5. Contrast-enhanced CT images of Case 7 at different stages. | | |

Case 8

A 52-year-old male with a history of hepatitis B was diagnosed with unresectable HCC in the left hepatic lobe, accompanied by a tumor thrombus in the middle hepatic vein. AFP was within the normal range. (A, B) Pretreatment contrast-enhanced MR showed HCC lesions in segment IV, with a tumor thrombus in the middle hepatic vein. (C, D) After three sessions of bHAIC, re-examination with contrast-enhanced MR showed a significant reduction in lesion size, the disappearance of tumor enhancement, and regression of the tumor thrombus. According to the mRECIST criteria, the therapeutic response was assessed as CR.

| 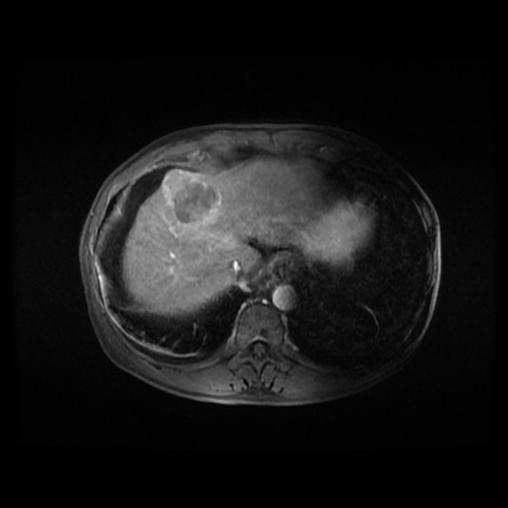 | 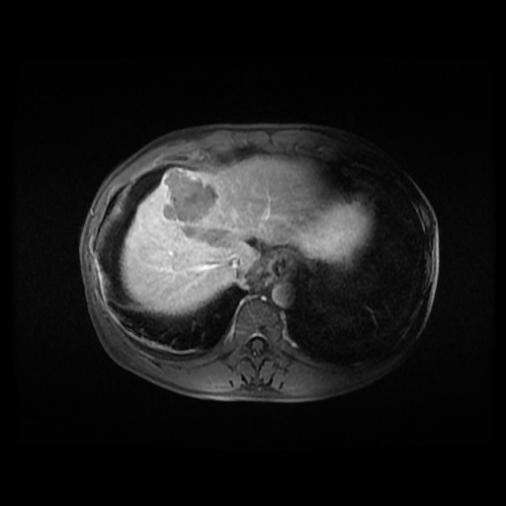 |
| --- | --- |
| A | B |
| 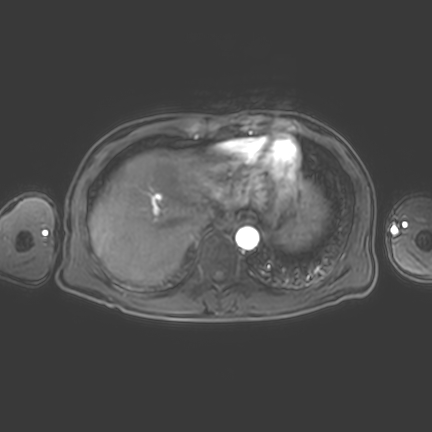 | 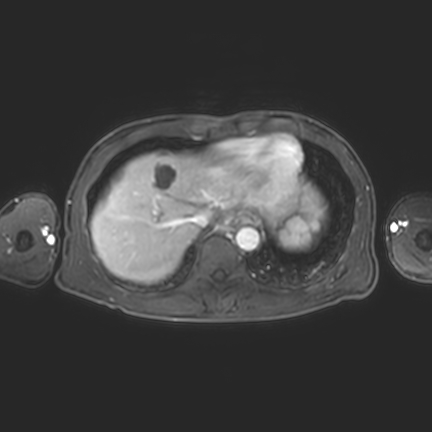 |
| C | D |
| Supplemental Figure 6. Contrast-enhanced CT images of Case 8 at different stages. | |

Case 9

A 70-year-old male with a history of hepatitis C was diagnosed with multiple scattered HCC in the right hepatic lobe, accompanied by Vp2-type portal vein tumor thrombus, with AFP level at 59,238 IU/mL (reference range: 0–5.8 IU/mL). (A, B) Pretreatment contrast-enhanced CT showed multiple HCC lesions in segments V, VI, VII, and VIII, with significant arterial phase enhancement. (C, D) After three sessions of bHAIC-FO treatment, the last follow-up post-bHAIC revealed a significant reduction in the lesion size and the disappearance of tumor enhancement. According to the mRECIST criteria, the therapeutic effect was assessed as CR.

| 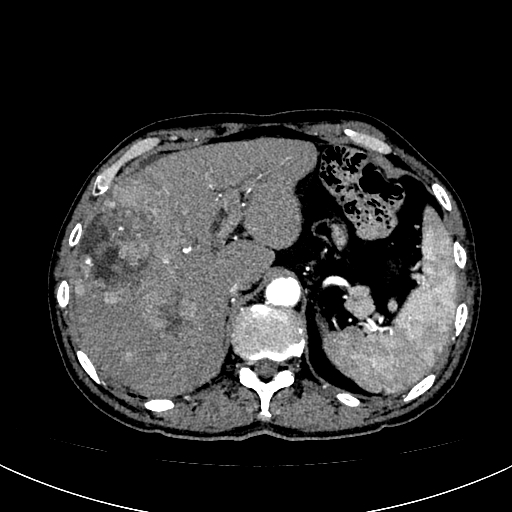 | 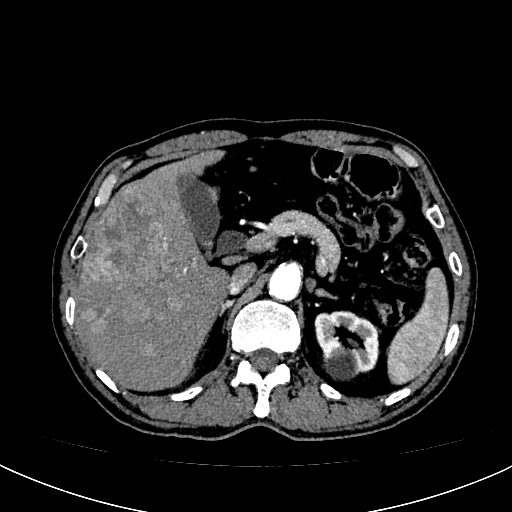 |
| --- | --- |
| A | B |
| 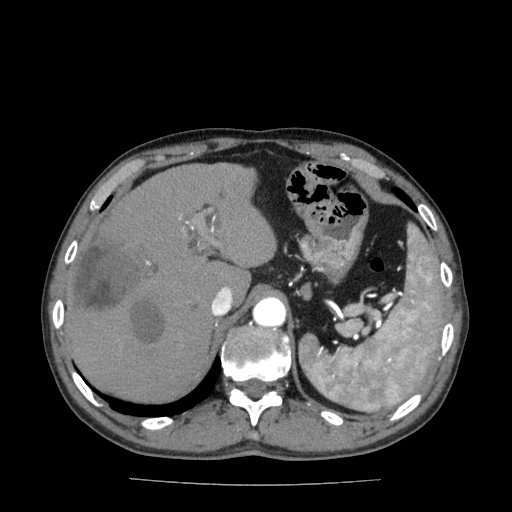 | 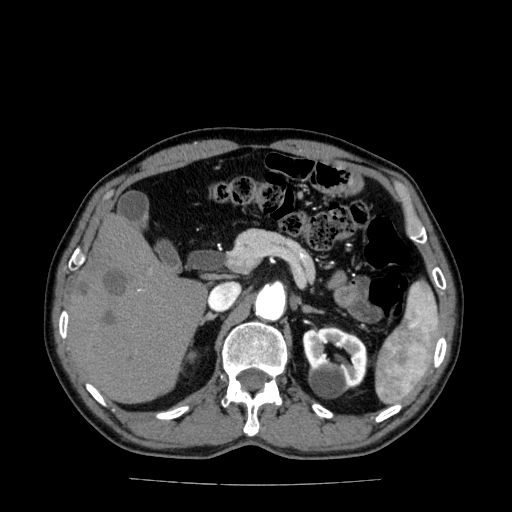 |
| C | D |
| Supplemental Figure 7. Contrast-enhanced CT images of Case 9 at different stages. | |

Case 10

A 51-year-old male with a history of hepatitis B was diagnosed with a large HCC accompanied by tumor thrombus in the middle hepatic vein, with an AFP level at 916 IU/mL (reference range: 0–5.8 IU/mL). (A, B) Pretreatment contrast-enhanced CT showed a large HCC lesion in segments V and VIII, with significant arterial phase enhancement. (C, D) After one session of bHAIC-FO and one session of conventional HAIC, re-examination with contrast-enhanced CT showed a significant reduction in lesion size and disappearance of tumor enhancement. According to the mRECIST criteria, the therapeutic effect was assessed as CR.

| 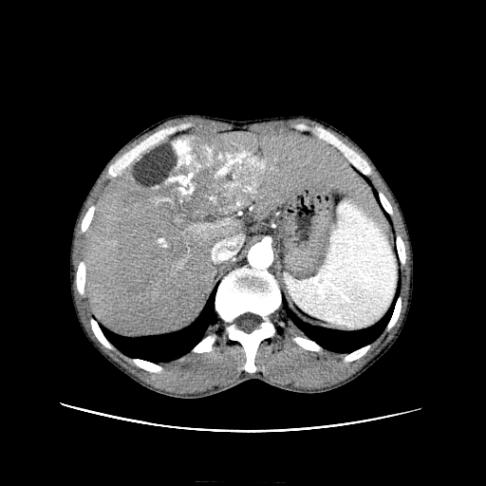 | 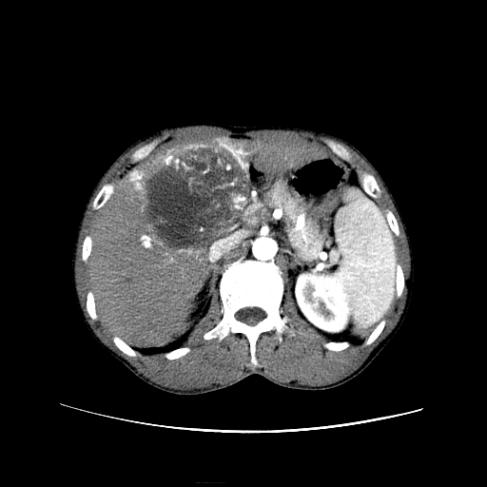 |
| --- | --- |
| A | B |
| 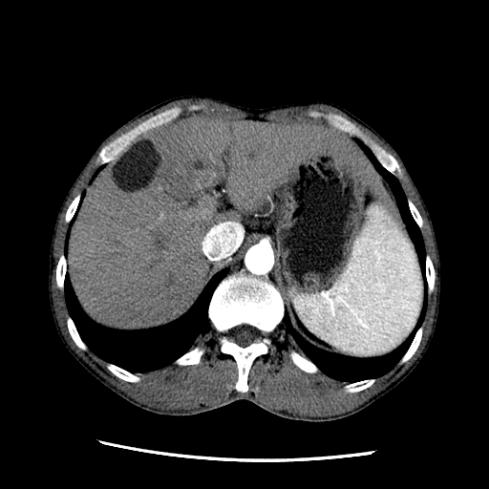 | 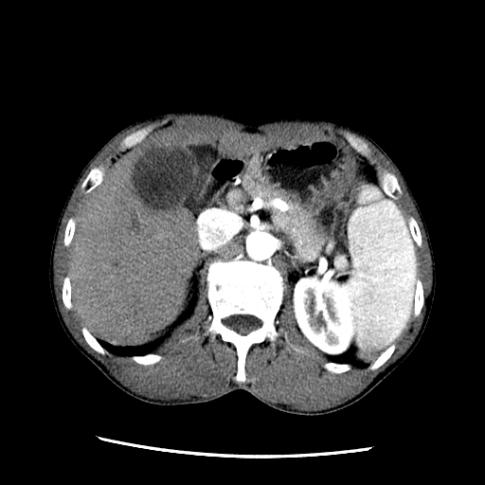 |
| C | D |
| Supplemental Figure 8. Contrast-enhanced CT images of Case 10 at different stages. | |

Case 11

A 48-year-old male with a history of hepatitis B was diagnosed with a large HCC in the right hepatic lobe, accompanied by tumor thrombus in the middle hepatic vein, with AFP level at IU/mL (reference range 0–5.8 IU/mL). (A, B) Pretreatment contrast-enhanced CT revealed a large HCC lesion in segments V, VI, VII, and VIII, with significant arterial phase enhancement. (C, D) After one session of bHAIC-FO and one session of conventional HAIC, re-examination with contrast-enhanced CT showed a significant reduction in the lesion size and disappearance of tumor enhancement. According to the mRECIST criteria, the therapeutic effect was assessed as CR.

| 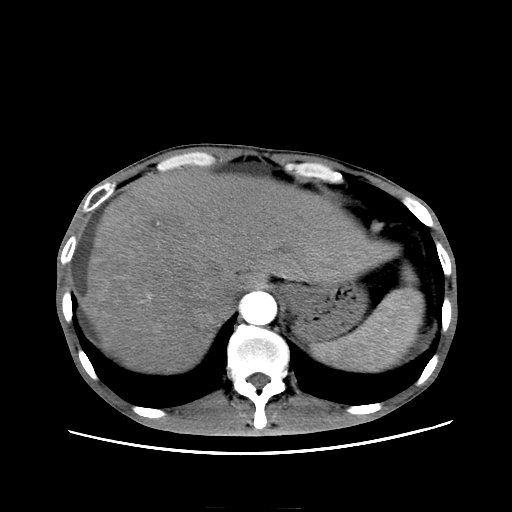 | 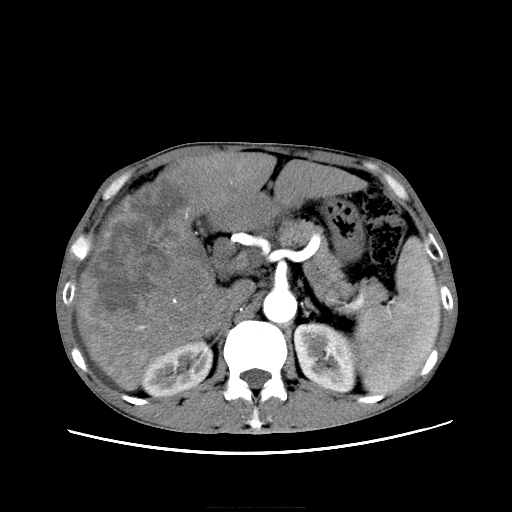 |
| --- | --- |
| A | B |
| 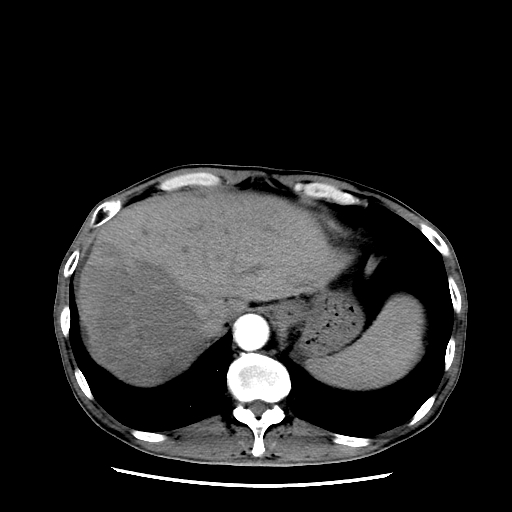 | 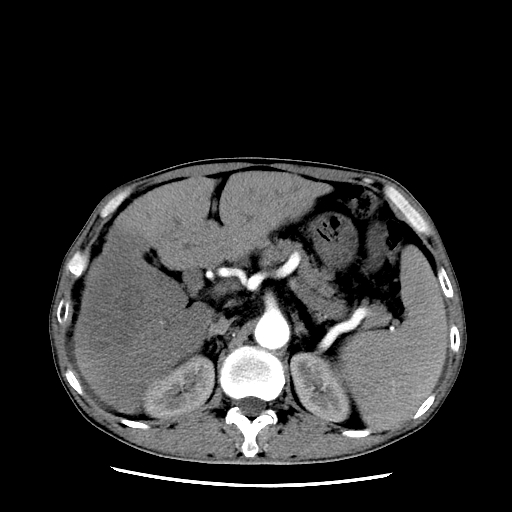 |
| C | D |
| Supplemental Figure 9. Contrast-enhanced CT images of Case 11 at different stages. | |

Case 12

A 55-year-old male with a history of hepatitis B was diagnosed with a large HCC in the left hepatic lobe, accompanied by Vp4-type portal vein tumor thrombus, with an AFP level at 1544 IU/mL (reference range: 0–5.8 IU/mL). (A, B) Pretreatment contrast-enhanced CT showed HCC lesions in segments II, III, and IV, with tumor thrombus in the left branches of the portal vein and the main trunk. (C, D) After three sessions of bHAIC-FO, re-examination with contrast-enhanced MR showed a slight reduction in the lesion size, along with the complete disappearance of tumor and portal vein thrombus enhancement. According to the mRECIST criteria, the therapeutic effect was assessed as CR.

| 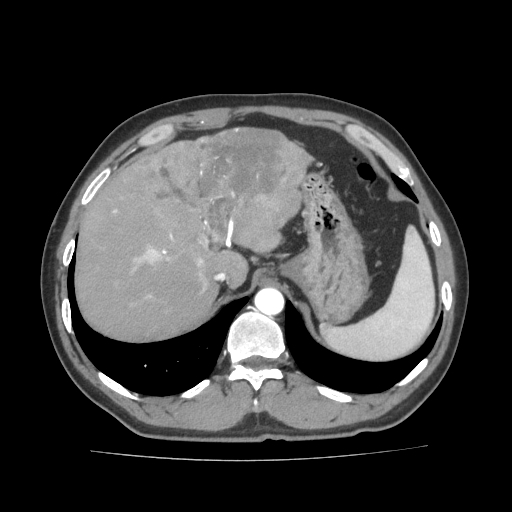 | 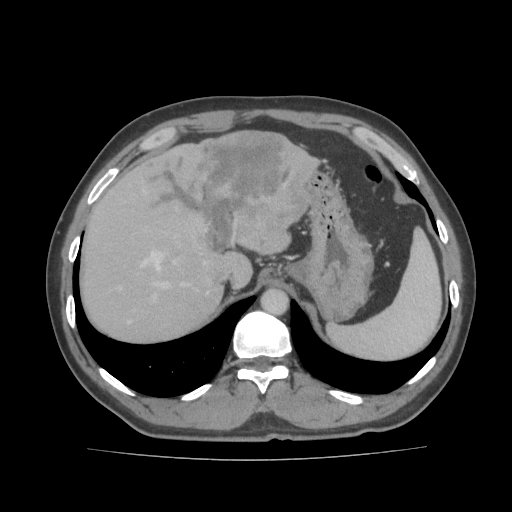 |
| --- | --- |
| A | B |
| 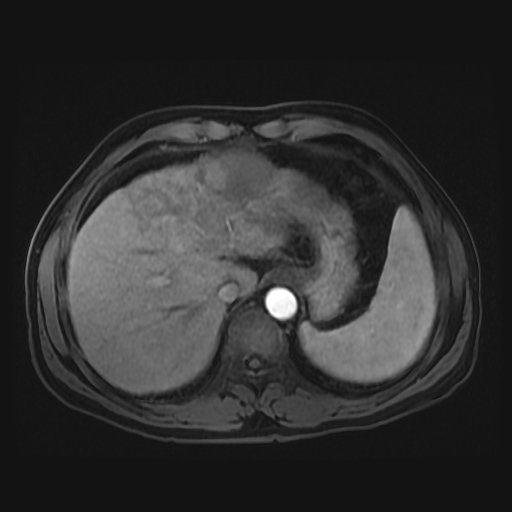 | 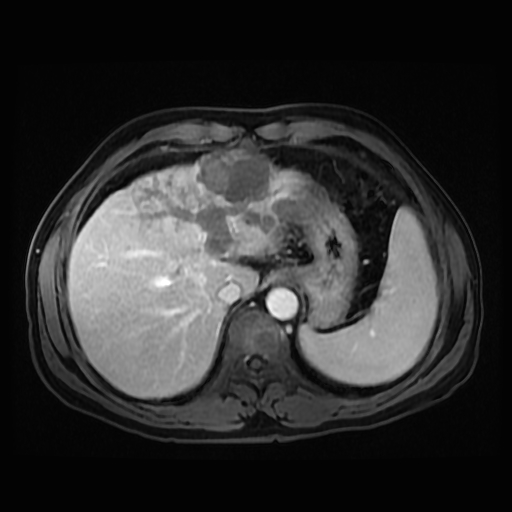 |
| C | D |
| Supplemental Figure 10. Contrast-enhanced CT images of Case 12 at different stages. | |

Case 13

A 53-year-old male with a history of hepatitis B was diagnosed with multiple scattered HCC lesions in segments V, VI, VII, and VIII, accompanied by Vp4-type portal vein tumor thrombus in the right branches of the portal vein and the main trunk, with an AFP level at 36 IU/mL (reference range: 0–5.8 IU/mL). (A, B, C) Pretreatment contrast-enhanced MR scan revealed multiple scattered HCC lesions in the right lobe of the liver, which exhibited enhancement during the late arterial phase. (D, E, F) After two sessions of bHAIC-FO, re-examination with contrast-enhanced MR showed the complete disappearance of tumor and portal vein thrombus enhancement. According to the mRECIST criteria, the therapeutic effect was assessed as CR.

| 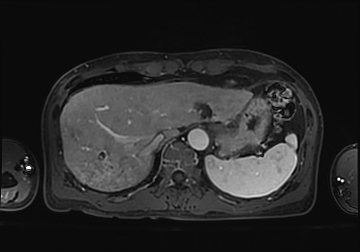 | 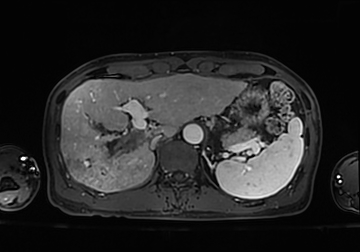 | 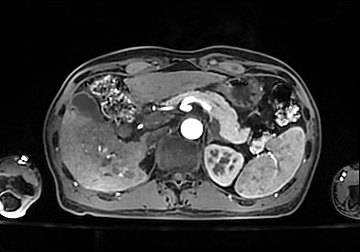 |
| --- | --- | --- |
| A | B | C |
| 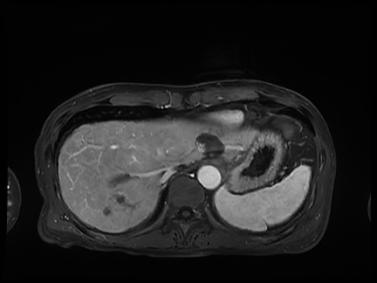 | 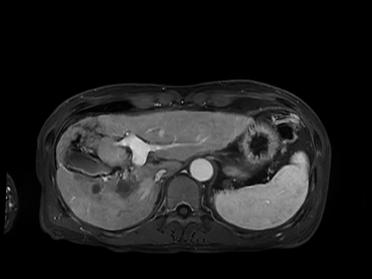 | 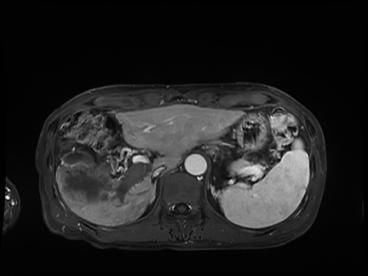 |
| D | E | F |
| Supplemental Figure 11. Contrast-enhanced CT images of Case 13 at different stages. | | |

Case 14

A 60-year-old male with a history of hepatitis B was diagnosed with multiple scattered HCC lesions in segments III, IV, V, VI, VII, and VIII, with an AFP level at 28.8 IU/mL (reference range: 0–5.8 IU/mL). (A, B) Pretreatment contrast-enhanced MR scan revealed multiple scattered HCC lesions in both the left and right liver lobes, which exhibited enhancement during the arterial phase. (C, D) After one session of bHAIC-FO and one session of TACE, re-examination with contrast-enhanced MR showed the complete disappearance of tumor enhancement. According to the mRECIST criteria, the therapeutic effect was assessed as CR.

| 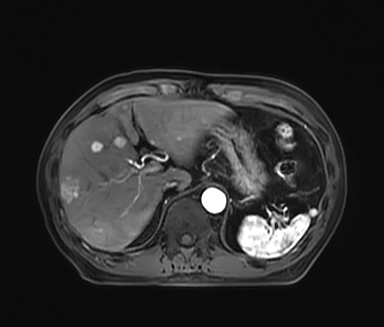 | 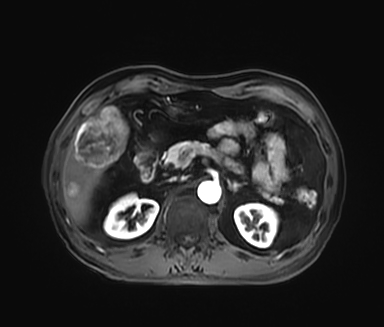 |
| --- | --- |
| A | B |
| 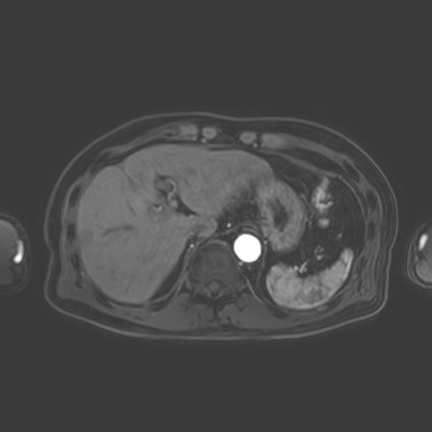 | 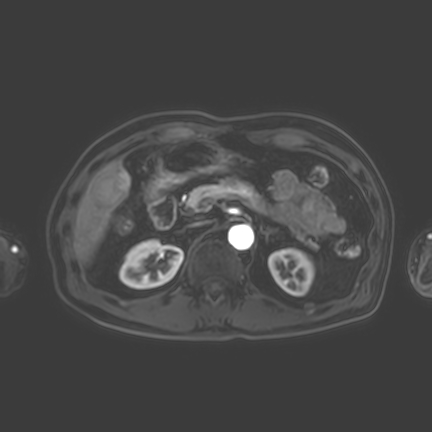 |
| C | D |
| Supplemental Figure 12. Contrast-enhanced CT images of Case 14 at different stages. | |

Case 15

See Case 2 in Supplemental Material 3.

Supplemental Material 3

Clinical data of 2 suspected cases of Tumor Lysis Syndrome (TLS)

Case 1

**Patient:** Male, 63 years old.

**Medical history:** Chronic hepatitis B

**Present condition:** Giant hepatocellular carcinoma (HCC) involving both the left and right lobes of the liver, accompanied by Vp2-type tumor thrombus. BCLC Stage: C; Child-Pugh Score: 6; Performance Status: 1; Alpha-Fetoprotein (AFP): 38.9 IU/mL (reference range 0–5.8 IU/mL).

**bHAIC drug infusion protocol:** oxaliplatin (110 mg/m^2^, arterial infusion over 2 hours), leucovorin (300 mg/m^2^, intravenous infusion over 2 hours), and fluorouracil (1100 mg/m^2^, continuous arterial infusion over 24 hours).

**Post-bHAIC management for potential TLS:** For patients with a tumor volume exceeding 50% of the liver volume, close monitoring of biochemical liver function, renal function, electrolytes, and complete blood count is required following the end of drug infusion. In this case, laboratory tests showed an elevated AST level of 502 IU/L six hours post-bHAIC and 2631 IU/L twelve hours post-bHAIC. The liver CT scan showed extensive low-density changes in the intrahepatic lesions, suggesting widespread tumor necrosis. Continuous Renal Replacement Therapy (CRRT) was immediately initiated for 32 hours. By the 8^th^ day post-bHAIC, AST had decreased to 103 IU/L, and all other laboratory indicators had significantly improved (Supplemental Table 1), allowing the patient to be discharged.

**Follow-up:** The patient underwent bHAIC treatment three times. According to the mRECIST criteria, the first efficacy evaluation was Partial Response (PR), and the final efficacy evaluation was PR 29 weeks post-bHAIC treatment (Supplemental Figure 13). The last follow-up showed AFP: 3.53 IU/mL.

**Supplemental Table 1.** Laboratory test indicators for patients undergoing bHAIC (Case 1).

|  | **AST** | **ALT** | **TBIL** | **CRE** | **WBC** | **N%** |
| --- | --- | --- | --- | --- | --- | --- |
| Pre-bHAIC | 158 IU/L | 85 IU/L | 31.3 μmol/L | 64 μmol/L | 6.31×10^9^/L | 54.3% |
| **Post-bHAIC** | | | | | | |
| 6 hours post-bHAIC | 502 IU/L | 138 IU/L | 23.4 μmol/L | 65 μmol/L | 7.0×10^9^/L | 86.0% |
| 12 hours post-bHAIC (initiate CRRT) | 2631 IU/L | 748 IU/L | 30.7 μmol/L | 62 μmol/L | 11.52×10^9^/L | 92.3% |
| 6 days post-bHAIC | 163 IU/L | 547 IU/L | 23.9 μmol/L | 70 μmol/L | 15.9×10^9^/L | 81.1% |
| 8 days post-bHAIC | 103 IU/L | 281 IU/L | 30.9 μmol/L | 73 μmol/L | 8.86×10^9^/L | 78.1% |

**Abbreviations:** ALT, alanine transaminase; AST, aspartate transaminase; CRE, creatinine; N%, neutrophil percentage; TBIL, total bilirubin; WBC, white blood cell count.

| 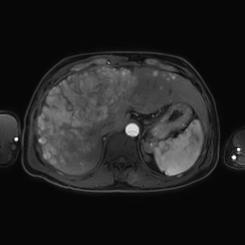 | 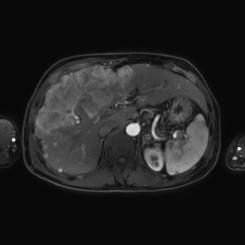 |
| --- | --- |
| A | B |
| 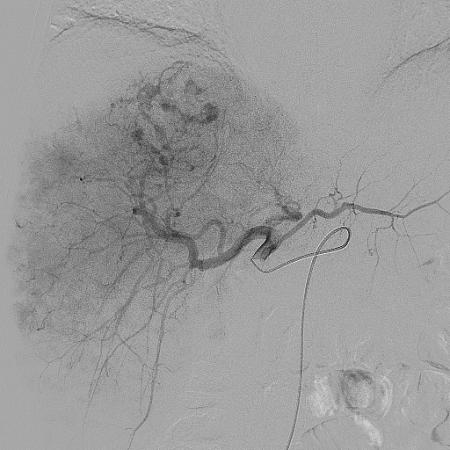 | 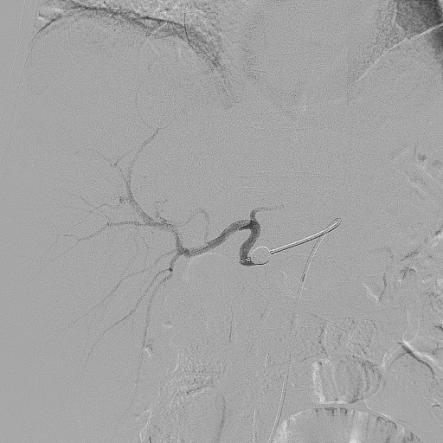 |
| C | D |
| 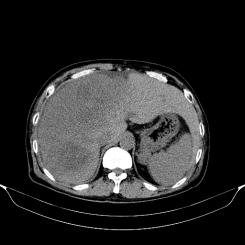 | 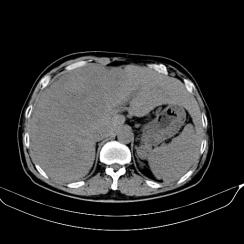 |
| E | F |
| 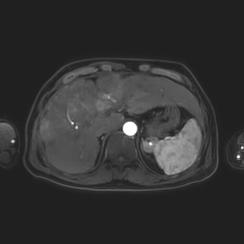 | 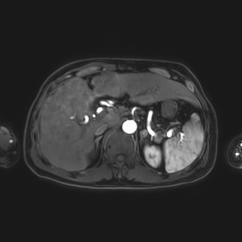 |
| G | H |

**Supplemental Figure 13.** Imaging data of a 63-year-old male with massive HCC who underwent bHAIC treatment. (A, B) Contrast-enhanced MR shows a giant HCC involving both the left and right lobes of the liver, with a cumulative extent exceeding 50% of the liver volume. (C, D) Selective digital subtraction angiogram (DSA) and balloon catheter hepatic artery occlusion images. (E, F) Post-bHAIC abdominal plain CT scan (E) compared to the pre-bHAIC baseline scan (F), the density of the lesions has markedly decreased. (G, H) The final efficacy evaluation was PR according to the mRECIST criteria 29 weeks post-bHAIC treatment.

Case 2

**Patient:** Male, 61 years old.

**Medical history:** Hepatitis B-related cirrhosis; mild renal dysfunction with a serum creatinine level of 145 μmol/L.

**Procedure history:** A transjugular intrahepatic portosystemic shunt (TIPS) procedure was performed five years ago to manage portal hypertension-induced gastrointestinal bleeding.

**Present condition:** Massive hepatocellular carcinoma (HCC) in the left lobe of the liver, accompanied by Vp3-type tumor thrombus. BCLC Stage: C; Child-Pugh Classification: Class B (Score 7); Performance Status: 2; Alpha-Fetoprotein (AFP): 30297.7 IU/mL (reference range: 0–5.8 IU/mL).

**bHAIC drug infusion protocol:** oxaliplatin (110 mg/m^2^, arterial infusion over 2 hours), leucovorin (300 mg/m^2^, intravenous infusion over 2 hours), and fluorouracil (1100 mg/m^2^, continuous arterial infusion over 24 hours).

**Post-bHAIC management for potential TLS:** For patients with a tumor volume exceeding 50% of the liver volume, close monitoring of biochemical liver function, renal function, electrolytes, and complete blood count is required following the end of drug infusion. In this case, six hours post-bHAIC, laboratory tests showed an elevated AST level of 616 IU/L. Given the patient's mild renal insufficiency prior to bHAIC, a second test was conducted three hours later, showing an AST level of 748 IU/L. A liver CT scan revealed a reduction in the density of the lesion in the left lobe compared to pre-bHAIC levels. Continuous Renal Replacement Therapy (CRRT) was immediately initiated for 24 hours. By the fifth day post-bHAIC, AST had decreased to 172 IU/L, and all other laboratory indicators had significantly improved (Supplemental Table 2), allowing the patient to be discharged.

**Follow-up:** The patient underwent post-bHAIC follow-up twice. According to the mRECIST criteria, the treatment efficacy was determined to be partial response (PR) at five weeks post-bHAIC, and complete response (CR) at nine weeks (Supplemental Figure 14). The last follow-up showed AFP: 13.3 IU/mL.

**Supplemental Table 2.** Laboratory Test Indicators for Patients Undergoing bHAIC (Case 2).

|  | **AST** | **ALT** | **TBIL** | **CRE** | **WBC** | **N%** |
| --- | --- | --- | --- | --- | --- | --- |
| Pre-bHAIC | 98 IU/L | 25 IU/L | 22.9 μmol/L | 145 μmol/L | 4.98×10^9^/L | 65.9% |
| **Post-bHAIC** | | | | | | |
| 6 hours post-bHAIC | 616 IU/L | 89 IU/L | 29.5 μmol/L | 191 μmol/L | 9.61×10^9^/L | 90.3% |
| 9 hours post-bHAIC (initiate CRRT) | 748 IU/L | 103 IU/L | 19.8 μmol/L | 194 μmol/L | 12.08×10^9^/L | 93.9% |
| 5 days post-bHAIC | 172 IU/L | 42 IU/L | 39.0 μmol/L | 163 μmol/L | 5.92×10^9^/L | 69.5% |

**Abbreviations:** ALT, alanine transaminase; AST, aspartate transaminase; CRE, creatinine; N%, neutrophil percentage; TBIL, total bilirubin; WBC, white blood cell count.

| 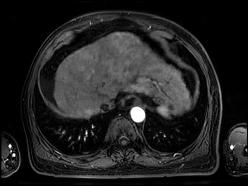 | 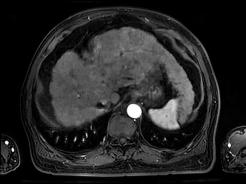 |
| --- | --- |
| A | B |
| 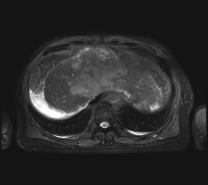 | 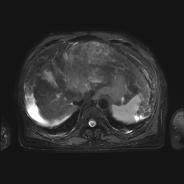 |
| C | D |
| 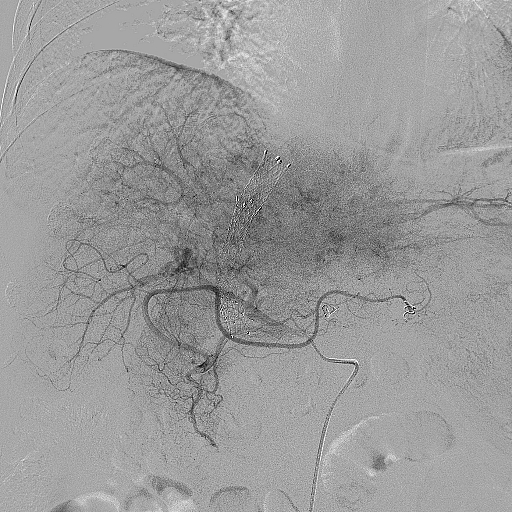 | 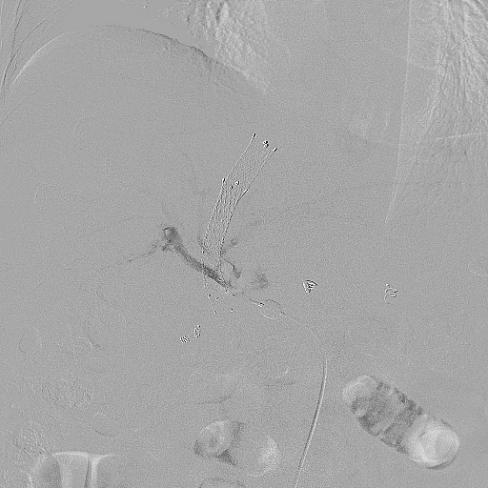 |
| E | F |
| 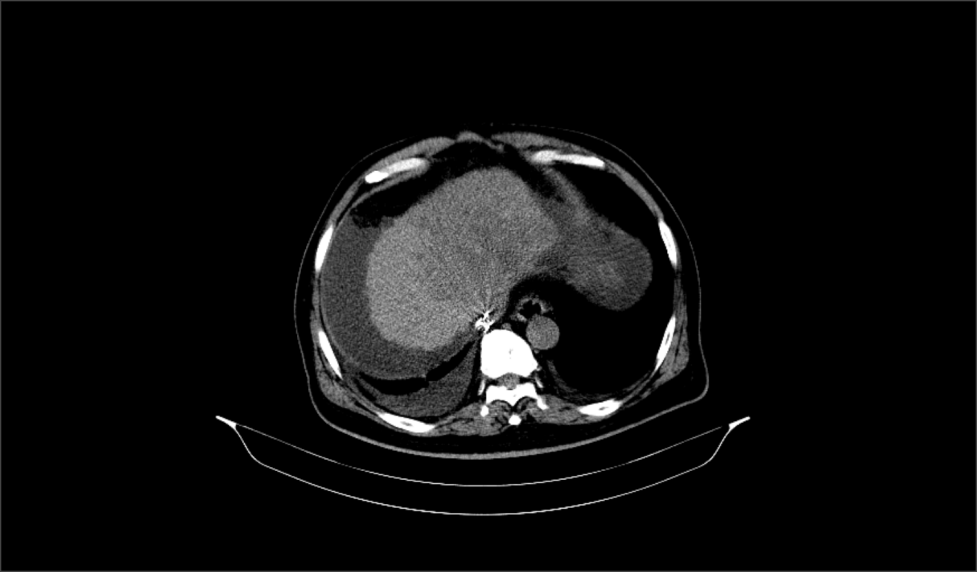 | 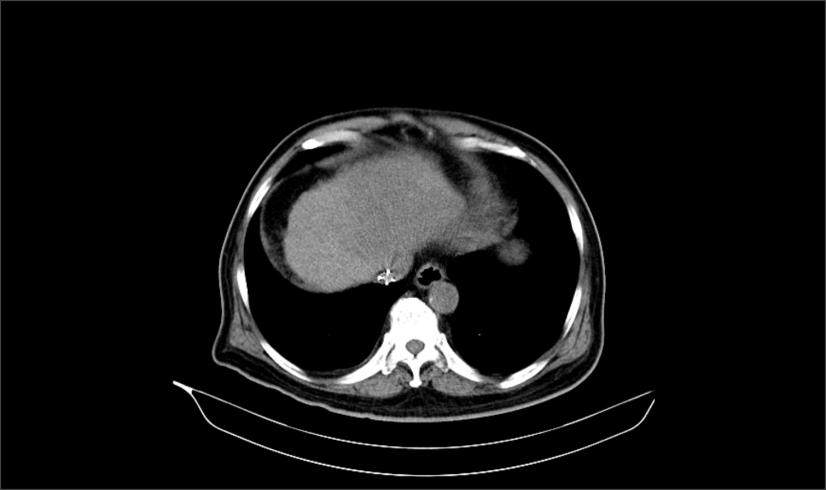 |
| G | H |
| 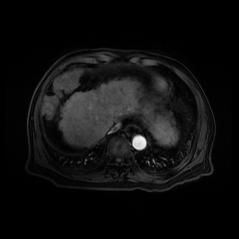 | 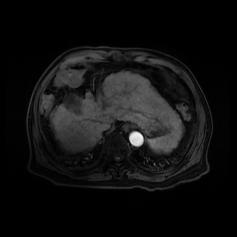 |
| I | J |
| 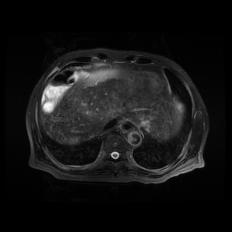 | 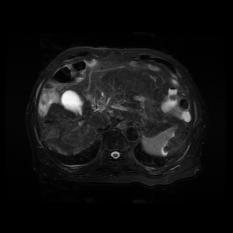 |
| K | L |

**Supplemental Figure 14.** Imaging data of a 61-year-old man with unresectable HCC who underwent bHAIC treatment. (A–D) Contrast-enhanced MR (A, B) and T2-weighted sequences (C, D) show a smaller right lobe of the liver and a massive HCC in the left lobe, accompanied by a Vp3-type tumor thrombus in the left portal vein branch. (E, F) Selective digital subtraction angiogram (DSA) and balloon catheter hepatic artery occlusion images. (G, H) Post-bHAIC abdominal plain CT scan (G) compared to the pre-bHAIC baseline scan (H), the density of the lesions has a markedly decreased. (I, J, K, L) The lesion enhancement has completely disappeared, and the signal intensity of the lesion on T2-weighted sequences has approached normalization 9 weeks after bHAIC.
